# Inhibition of Hippo Signaling Through Ablation of Lats1 and Lats2 Protects Against Cognitive Decline in 5xFAD Mice via Increasing Neuronal Resilience Against Ferroptosis

**DOI:** 10.1111/acel.70218

**Published:** 2025-09-09

**Authors:** Robert C. Evans, Nawab John Dar, Liuji Chen, Ren Na, Jason C. O'Connor, Jing Jiang, Siyuan Zheng, Qitao Ran

**Affiliations:** ^1^ Department of Cell Systems & Anatomy University of Texas Health San Antonio San Antonio Texas USA; ^2^ Department of Pharmacology University of Texas Health San Antonio San Antonio Texas USA; ^3^ Research Service South Texas Veterans Health Care System San Antonio Texas USA; ^4^ Greehey Children's Cancer Research Center University of Texas Health San Antonio San Antonio Texas USA; ^5^ The Biggs Institute for Alzheimer's and Neurodegenerative Diseases University of Texas Health San Antonio San Antonio Texas USA

**Keywords:** 5xFAD mice, Alzheimer's disease, ferroptosis, Hippo signaling, lipid peroxidation

## Abstract

The Hippo signaling pathway is a key regulator of cell growth and cell survival, and hyperactivation of the Hippo pathway has been implicated in neurodegenerative diseases such as Huntington's disease. However, the role of Hippo signaling in Alzheimer's disease (AD) remains unclear. We observed that hyperactivation of Hippo signaling occurred in the AD model 5xFAD mice. To determine how inhibition of Hippo signaling might affect disease pathogenesis, we generated 5xFAD mice with conditional neuronal ablation of Lats1 and Lats2, the gatekeepers of Hippo signaling activity. Our results indicated that 5xFAD mice with ablation of Lats1 and Lats2 were protected against cognitive decline compared with control 5xFAD mice, and this protection was correlated with a marked reduction in neurodegeneration. Interestingly, primary culture neurons with ablation of Lats1 and Lats2 had significantly increased survival following treatment with chemical inducers of ferroptosis and exhibited reduced lipid peroxidation, the driving force of ferroptotic cell death. Moreover, 5xFAD mice with ablation of Lats1 and Lats2 showed reduced lipid peroxidation, and transcriptomic analysis revealed that 5xFAD mice with ablation of Lats1 and Lats2 had enriched metabolic pathways associated with ferroptosis. These results indicate that inhibition of Hippo signaling activity confers neural protection in 5xFAD mice by augmenting resilience against ferroptosis.

## Graphical abstract

5xFAD mice exhibited abnormal activation of Hippo signaling. Ablation of Lats1 and Lats2 in 5xFAD mice to inhibit Hippo signaling resulted in augmented cognition and ameliorated neurodegeneration, which were mediated by increased neuronal resilience against ferroptosis.

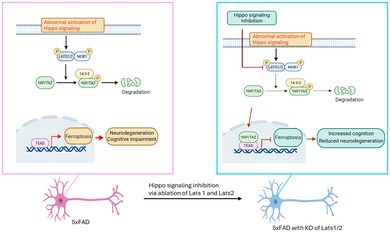

## 1 Introduction

Alzheimer's Disease (AD) is the leading cause of dementia, estimated to be responsible for 50%–60% of all cases (Blennow et al. 2006). Despite recent progress in therapy development (Self and Holtzman 2023; Perneczky et al. 2024), efficacious treatments that can significantly slow the progression of AD are still lacking. The primary pathological markers/features of AD include the presence of both amyloid beta (Aβ) plaques and tau neurofibrillary tangles, as well as rampant neuroinflammation. A plethora of those and other pathogenic events precipitate the degeneration of synapses and death of neurons, which is ultimately the cause of key symptoms of AD including memory loss and cognitive impairment (Gomez‐Isla et al. 1997). Because of this, it has long been proposed that neuroprotective treatments aimed to improve health and prevent death of neurons would be beneficial for managing AD symptoms and slowing disease progression (Donev et al. 2009).

The Hippo signaling pathway is a cellular mechanism that regulates cell growth, proliferation, and survival (Kim and Jho 2014; Meng et al. 2016). Hippo signaling negatively regulates the activity of transcriptional coactivators Yes‐associated protein 1 (YAP) and transcriptional coactivator with PDZ‐binding motif (TAZ). When Hippo signaling activity is low or inhibited, YAP and TAZ enter the nucleus and form a complex with TEAD1‐4, thereby allowing for the expression of genes that promote growth and proliferation. The Hippo signaling pathway also regulates cell survival, as increased YAP activity has been shown to suppress apoptosis (Kim and Jho 2014; Zhang et al. 2018). Recent evidence has further implicated this pathway in the regulation of ferroptosis, an iron‐dependent and oxidative mode of cell death (Yang et al. 2019; Wu et al. 2019).

The Hippo signaling pathway plays a critical role in neural development. In the developing brain, the Hippo signaling pathway regulates the size of the neural progenitor pool and ensures proper neurogenesis and the formation of neural circuits (Sahu and Mondal 2020a). Although dysregulation of Hippo signaling activity is identified in several neurodevelopmental conditions (Sahu and Mondal 2020b; Park et al. 2016; Sukumaran et al. 2017; Williamson et al. 2014), the Hippo signaling pathway has long been thought to play a rather limited role in adult neurons. However, recent reports suggested that Hippo signaling hyperactivity may be associated with neurodegenerative conditions (Sahu and Mondal 2020a; Fu et al. 2022). For instance, Hippo signaling activity was reported to be elevated in the cortical neurons of both Huntington's disease (HD) patients and mouse models (Mueller et al. 2018). Hippo signaling pathway dysregulation was also implicated as a potential contributing factor in AD‐associated pathogenesis (Liu et al. 2018; Xu et al. 2018; Bakshi et al. 2018). However, the direct role of altered Hippo signaling activity in AD pathogenesis remains largely unknown.

Large tumor suppressor 1 (LATS1) and large tumor suppressor 2 (LATS2) are transmembrane kinases critical for regulating Hippo signaling activity (Kim and Jho 2014). Upon activation of upstream Hippo regulators such as MST1 and MST2, a cascade of kinase signaling events leads to phosphorylation and activation of LATS1 and LATS2, which in turn phosphorylate YAP and TAZ proteins, leading to increased cytoplasmic retention and/or degradation of YAP and TAZ and subsequent inhibition of downstream Hippo activity (Liu et al. 2019).

In this study, we assessed the status of downstream activation of Hippo pathway activity in 5xFAD mice, a widely used AD mouse model in which the expression of 5 FAD mutations leads to a high load of Aβ, cognitive impairment, and robust neurodegeneration (Oakley et al. 2006; Ohno et al. 2007). We further evaluated the effects of Hippo signaling inhibition via ablation of both Lats1 and Lats2 genes on the development of symptoms and pathology in the 5xFAD mouse model. Our results indicated that the Hippo pathway was hyperactivated in 5xFAD mice and that downstream Hippo signaling inhibition in neurons through ablation of Lats1 and Lats2 resulted in improved cognition and reduced neurodegeneration in 5xFAD mice. Our results further demonstrated that the beneficial effect of Hippo activity inhibition was mediated by increased neuronal resilience against ferroptosis.

## 2 Results

### 2.1 Abnormal Activation of Hippo Signaling in 5xFAD Mice

**FIGURE 1:**
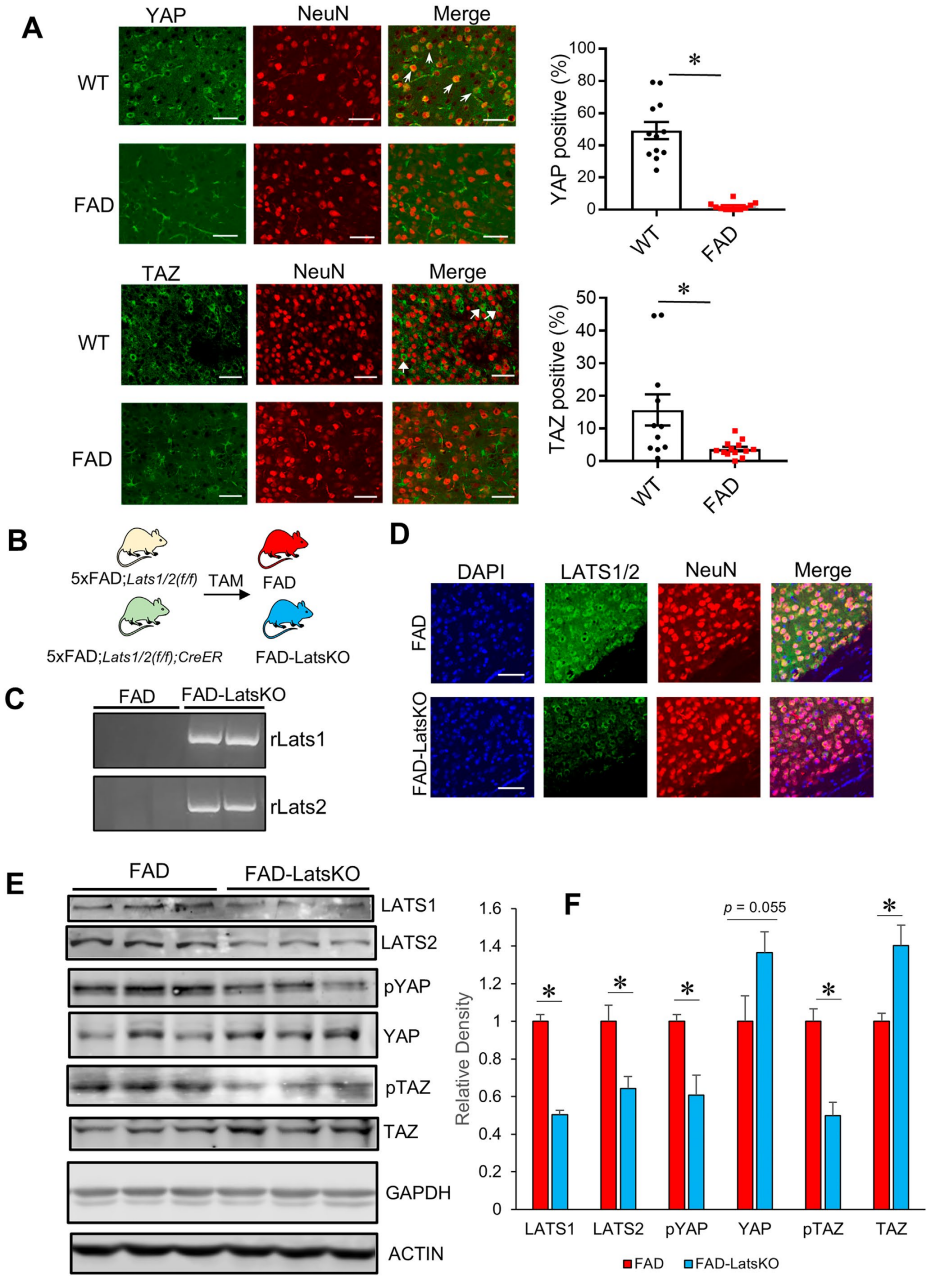
Reduced YAP and TAZ in 5xFAD mice and generation of 5xFAD mice with neuronal knockout of Lats1 and Lats2. (A) Representative images and quantified levels of YAP and TAZ positive neurons in cortices of WT and 5xFAD (FAD) mice. White arrows indicated YAP or TAZ positive neurons. *p < 0.05. (B) Scheme for generating 5xFAD mice with neuronal knockout (KO) of Lats1 and Lats2 (FAD‐LatsKO). (C) PCR gel images showing the presence of rLats1 and rLats2 deletion amplicons in samples from FAD‐LatsKO mice. (D) Representative images of LATS1/2‐stained cortical brain sections from FAD and FAD‐LatsKO mice. (E) Immunoblotting results showing Hippo pathway protein levels in the cortices of FAD and FAD‐LatsKO mice, with GAPDH and ACTIN used as loading controls. (F) Quantified results of Immunoblotting. *n* = 3 mice per condition. Error bars represent SEM. *p < 0.05.

Hyperactivation of Hippo signaling in neurons has been reported in diseases such as HD, and altered Hippo signaling was also implicated in AD (Xu et al. 2018; Tanaka et al. 2020). 5xFAD mouse is a widely used AD mouse model known to develop progressive cognitive impairments, Aβ pathology, and robust neurodegeneration (Forner et al. 2021; Jawhar et al. 2012; Schneider et al. 2014). To examine the status of Hippo signaling activity, we compared levels of YAP and TAZ in cortical neurons from 5xFAD mice and control WT mice (15 months of age). The brain sections were stained with antibodies specific for YAP or TAZ and co‐stained with an antibody for neural marker protein NeuN. As shown in Figure 1A, 5xFAD mice showed a reduced number of YAP‐positive neurons compared with age‐matched WT mice. Similarly, 5xFAD mice also showed a reduced number of TAZ‐positive neurons (Figure 1A). As the levels of YAP and TAZ are negatively regulated by Hippo signaling, the reduced numbers of YAP and TAZ positive neurons of 5xFAD mice indicated abnormal activation of Hippo signaling.

### 2.2 Generation of 5xFAD Mice With Conditional Knockout of LATS1 and LATS2 in Forebrain Neurons (FAD‐LatsKO)

To investigate the significance of abnormal activation of Hippo signaling activity in the pathogenesis of 5xFAD mice, we were interested in determining how inhibition of Hippo signaling in neurons might affect cognition and AD‐related pathology. LATS1 and LATS2 are the two regulators of YAP and TAZ activity. As ablation of both Lats1 and Lats2 genes is expected to result in Hippo signaling activity inhibition, we set out to generate a 5xFAD mouse model in which Lats1 and Lats2 could be ablated specifically in neurons. We cross‐bred 5xFAD mice with floxed Lats1 and Lats2 (Lats1/Lats2(f/f)) alleles and wild‐type mice with a tamoxifen (TAM) inducible Cre in forebrain and hippocampal excitatory neurons (Camk2α‐CreER). After several rounds of breeding, we obtained 5xFAD; Lats1/Lats2(f/f); Camk2α‐CreER mice. In these mice, ablation of both Lats1 and Lats2 in forebrain neurons should be subject to temporal regulation by treatment with TAM, thereby allowing ablation of Lats1 and Lats2 in animals at post‐developmental stages. This distinction is important given that dysregulation of Hippo signaling has been shown to cause neurodevelopmental abnormalities (Sahu and Mondal 2020b; Park et al. 2016; Sukumaran et al. 2017; Williamson et al. 2014).

To validate this model, we treated 5xFAD; Lats1/Lats2(f/f); Camk2α‐CreER mice (3 months of age) with TAM to induce Cre recombination and knockout (KO) of Lats1 and Lats2 (Figure 1B). To assess whether the KO of Lats1 and Lats2 was successful, we first tried to detect the presence of recombinant alleles of Lats1 and Lats2 (i.e., rLats1 and rLats2) in TAM‐treated 5xFAD; Lats1/Lats2(f/f); Camk2α‐CreER mice (referred to as FAD‐LatsKO mice). Figure 1C shows PCR gel images indicating the presence of deletion amplicons derived from rLats1 and rLats2 in DNA samples of cortices from FAD‐LatsKO mice but not in those from 5xFAD; Lats1/Lats2(f/f) mice without the Camk2α‐CreER transgene (control FAD mice).

We next assessed the protein levels of LATS1 and LATS2 in the cortices of FAD‐LatsKO and control FAD mice via immunofluorescence (IF) staining. Because of the high degree of sequence homology between LATS1 and LATS2 proteins, a polyclonal LATS2 antibody that also shows reactivity to LATS1 was used as a stand‐in for both proteins. Representative images of the antibody‐stained brain sections are shown in Figure 1D. A decrease in fluorescence signal can be observed in the cortical neurons of FAD‐LatsKO mice compared to FAD mice. KO of Lats1 and Lats2 and inhibition of downstream Hippo signaling activity in FAD‐LatsKO mice were further confirmed via immunoblotting. As shown in Figure 1E,F, FAD‐LatsKO mice showed reduced levels of LATS1 and LATS2 proteins compared to control FAD mice. LATS1 and LATS2 function as protein kinases that phosphorylate YAP and TAZ, making them incapable of entering the nucleus where they would form complexes with TEAD proteins to act as transcription cofactors, and instead tagging them for degradation and/or cytoplasmic retention (Kim and Jho 2014). Consistent with KO of LATS1 and LATS2, samples from FAD‐LatsKO mice showed significantly increased protein levels of total YAP and a trending increase in total TAZ, as well as decreased levels of phosphorylated YAP and TAZ (Figure 1D,E).

Inhibition of Hippo signaling activity has previously been found to be associated with abnormal growth and cancer (Harvey et al. 2013). To determine whether KO of Lats1 and Lats2 in mature cortical neurons causes increased cell growth and proliferation, we stained cortical sections from 10‐month‐old FAD‐LatsKO mice with Ki‐67, a marker of proliferation that has been used to detect neurogenesis and cancerous growth (Jaros et al. 1992; Kawasaki et al. 2001; Jin et al. 2006; Kee et al. 2002), and found no signs of increased proliferation or tumorigenesis as a result of KO of Lats1 and Lats2 (Figure S1).

### 2.3 FAD‐LatsKO Mice Exhibited Improved Cognition Compared to Control FAD Mice

**FIGURE 2:**
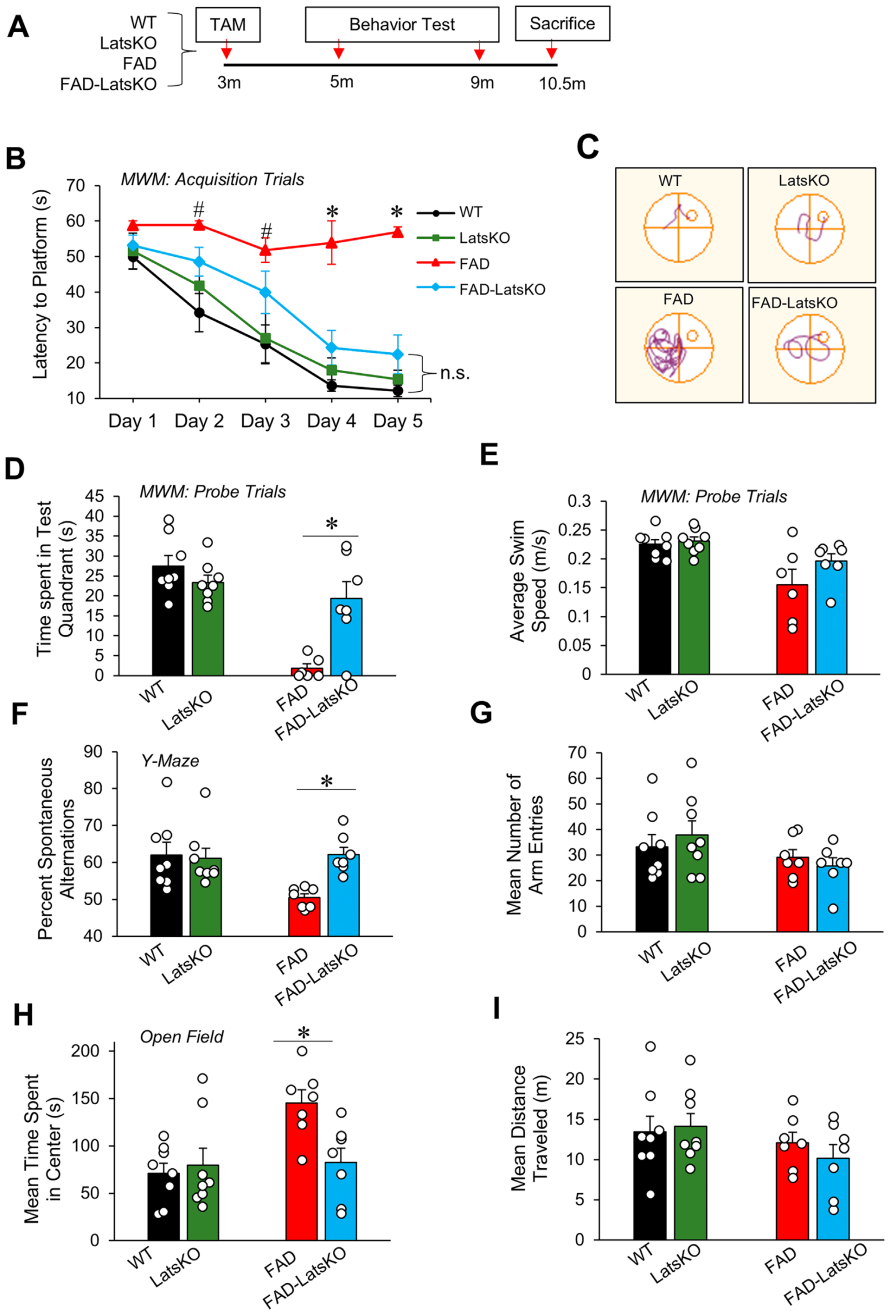
FAD mice with neuronal knockout of Lats1 and Lats2 exhibited improved cognition. (A) Scheme for assessing cognitive function of experimental groups. (B) Results of Morris water maze (MWM) acquisition trials at 9 months of age. #*p* < 0.05 between the FAD and both the WT and LatsKO groups, *p < 0.05 between the FAD and all three other groups. (C) Representative swim plots of acquisition trials on Day 5. (D) Results of MWM probe trials on Day 6. (E) The average swim speed during MWM probe trials. (F) Result of the Y‐Maze Task showing spontaneous alternations at 9 months of age. (G) The number of arm entries performed during the Y‐Maze Task. (H) Results of the Open Field Task showing time spent in the center of the arena at 9 months of age. (I) Mean distance traveled in the Open Field task. Error bars represent SEM. Mixed gender, *n* = 6–8 mice per group, *p < 0.05.

After confirming the efficacy of ablation of Lats1 and Lats2 to inhibit downstream Hippo signaling activity, we set out to determine how inhibition of Hippo signaling activity might affect cognition and AD‐related pathology in 5xFAD mice. To that end, we generated cohorts of 5xFAD and WT mice with or without conditional ablation of Lats1 and Lats2. Since cognitive impairments generally occur in 5xFAD mice around 4–6 months of age and become progressively worse as they age (Forner et al. 2021; Jawhar et al. 2012; Schneider et al. 2014), we treated the mice with TAM at 3 months of age, when normal brain development has occurred but no cognitive impairments are expected in 5xFAD mice. This treatment led to the establishment of four groups which are referred to hereafter as FAD‐LatsKO (5xFAD with Lats1 and Lats2 KnockOut), FAD (5xFAD control), LatsKO (Wildtype with Lats1 and Lats2 KnockOut), and WT (Wildtype control), respectively. The mice were subjected to a battery of behavioral tests at 5 months and 9 months of age to assess the effects of neuronal KO of Lats1 and Lats2 on AD‐associated cognitive decline (Figure 2A).

The Morris water maze (MWM) was used to evaluate differences in spatial learning and memory between the FAD and WT groups with or without neuronal KO of Lats1 and Lats2 (Vorhees and Williams 2006). In this task, cognitively normal mice will show a reduced latency to finding the hidden platform, known as escape latency, as the acquisition trials progress, and spend a greater proportion of time searching the correct quadrant during the probe trial. However, 5xFAD mice exhibit notably impaired spatial learning and memory (Schneider et al. 2014; Urano and Tohda 2010; Yang et al. 2015). The results from the 5‐month testing timepoint indicate that FAD mice performed slightly worse than the other groups, but the differences were relatively mild (Figure S2A–D). However, when tested again at the 9‐month timepoint, the FAD mice did not exhibit a noticeable improvement in their performance over the 5‐day acquisition trial period, indicating that they were unable to learn and recall the location of the hidden platform (Figure 2B, with representative swim plots shown in Figure 2C). In contrast, FAD‐LatsKO mice exhibited a reduction in escape latency that closely resembled the learning curves recorded for WT and LatsKO mice, and each of these three groups had a significantly lower mean escape latency on days 4 and 5 compared to FAD mice. In the probe trials, 9‐month‐old FAD mice spent significantly less time searching in the correct quadrant than all three other groups did (Figure 2D), while FAD‐LatsKO mice exhibited a similar proficiency in searching in the correct quadrant to that of WT and LatsKO mice. There were no significant differences between the FAD and FAD‐LatsKO groups in average swim velocity (Figure 2E), indicating that the MWM performance differences between FAD mice and FAD‐LatsKO mice were not caused by motor deficits.

We also used the Y‐Maze task to evaluate the working memory of our mouse groups. Mice with normal spatial memory will often perform a high number of spontaneous alternations compared to mice with impaired cognition, such as aged 5xFAD mice, which will often move back and forth between the arms they have most recently entered (Oakley et al. 2006; Ohno et al. 2007; Jeong et al. 2015). The results show that both FAD and FAD‐LatsKO mice did not perform a significantly lower percentage of spontaneous alternations compared to age‐matched WT and LatsKO mice at 5 months of age (Figure S2E). However, at 9 months of age, FAD mice had a significantly lower percentage of spontaneous alternations compared to both WT and LatsKO groups (Figure 2F). In contrast, FAD‐LatsKO mice did not perform significantly worse than WT or LatsKO mice and had a significantly higher percentage of spontaneous alternations than FAD mice, indicating intact short‐term spatial memory. Despite the marked difference in spontaneous alternations between FAD‐ and FAD‐LatsKO mice, there was no difference in the overall arm entries between the two groups (Figure 2G), indicating that locomotor activity, ability, or motivation did not contribute to the performance difference on the Y‐Maze task between FAD‐LatsKO mice and FAD mice.

Behavioral disinhibition, a tendency to become less concerned with certain actions and behaviors that would normally be recognized as dangerous or harmful, manifests in both AD patients and AD animal models (Jawhar et al. 2012; Devanand et al. 1992; Starkstein et al. 2004). To assess changes in disinhibitory behavior, we employed the Open Field test. In this test, control mice tend to spend a greater proportion of their time along the outer edge of the Open Field arena, while aged 5xFAD mice have been found to spend more time in the center of the arena (Forner et al. 2021; Jeong et al. 2015). At 5 months of age, there were no significant differences in the proportion of time spent near the arena's center between any of the four groups (Figure S2G). This is consistent with the findings of previous studies that indicate no behavioral disinhibition in young 5xFAD mice (Forner et al. 2021; Jawhar et al. 2012). However, when tested again at 9 months, there was a significant increase in the average amount of time spent in the center of the arena for FAD mice compared to WT and LatsKO mice (Figure 2H), indicating the development of behavioral disinhibition. However, this did not occur in FAD‐LatsKO mice, which spent a significantly lower amount of time in the center of the arena compared to FAD mice. No differences in the mean distance traveled were observed among the groups (Figure 2I), suggesting that the protective effect against behavioral disinhibition observed in FAD‐LatsKO mice was not due to locomotor activity differences.

### 2.4 Amyloid Beta Pathology Was Unaltered in FAD‐LatsKO Mice

**FIGURE 3:**
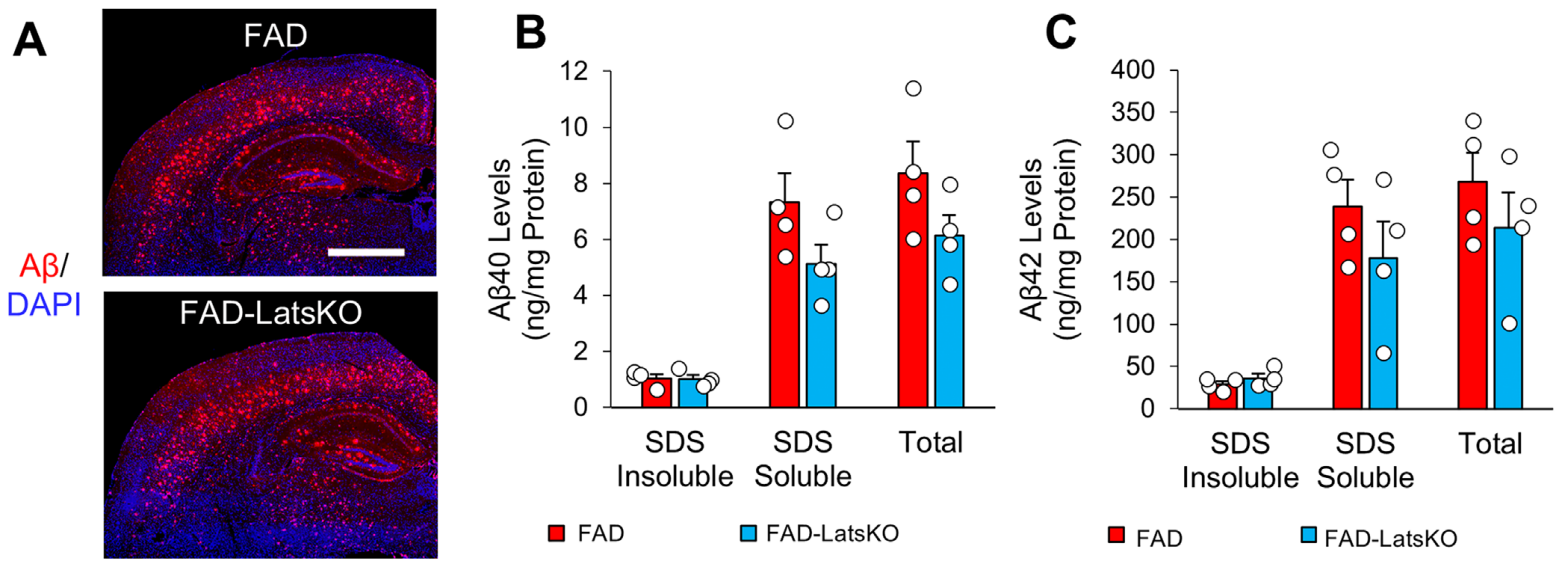
FAD mice with neuronal knockout of Lats1 and Lats2 showed no altered amyloid beta levels. (A) Representative images of brain sections from FAD and FAD‐LatsKO mice stained with an anti‐Aβ antibody and DAPI. Scale bar represents 1 mm. (B, C) SDS soluble, SDS insoluble, and total protein levels of Aβ40 (B) and Aβ42 (C) in the cortices of FAD and FAD‐LatsKO mice, as measured by ELISA assay (*n* = 4 mice per group). Error bars represent SEM.

After behavioral testing, the mice were sacrificed to obtain brain specimens for immunohistological and biochemical analyses. To determine whether inhibition of Hippo signaling might affect Aβ accumulation, which is a key hallmark of AD in human patients and 5xFAD mice, brain sections taken from FAD and FAD‐LatsKO mice were stained with an antibody specific to Aβ and imaged with confocal microscopy. Representative images from this immunofluorescence (IF) staining indicate that there were no obvious differences in Aβ burdens between FAD and FAD‐LatsKO mice (Figure 3A). We further compared Aβ burdens by quantifying Aβ levels. Cortical tissue samples taken from FAD and FAD‐LatsKO mice were processed as described in the Methods in order to generate SDS soluble and insoluble fractions. Commercial ELISA assay kits were then used to measure soluble and insoluble levels of Aβ40 and Aβ42, the two dominant Aβ species found in the brains of 5xFAD mice as well as AD patients (Chen et al. 2022; Eimer and Vassar 2013; Findeis 2007; Gu and Guo 2013). The results from these ELISA assays indicate that there were no significant differences in either Aβ40 or Aβ42 levels between FAD‐LatsKO mice and control FAD mice (Figure 3B,C).

### 2.5 FAD‐LatsKO Mice Exhibited Reduced Neurodegeneration

**FIGURE 4:**
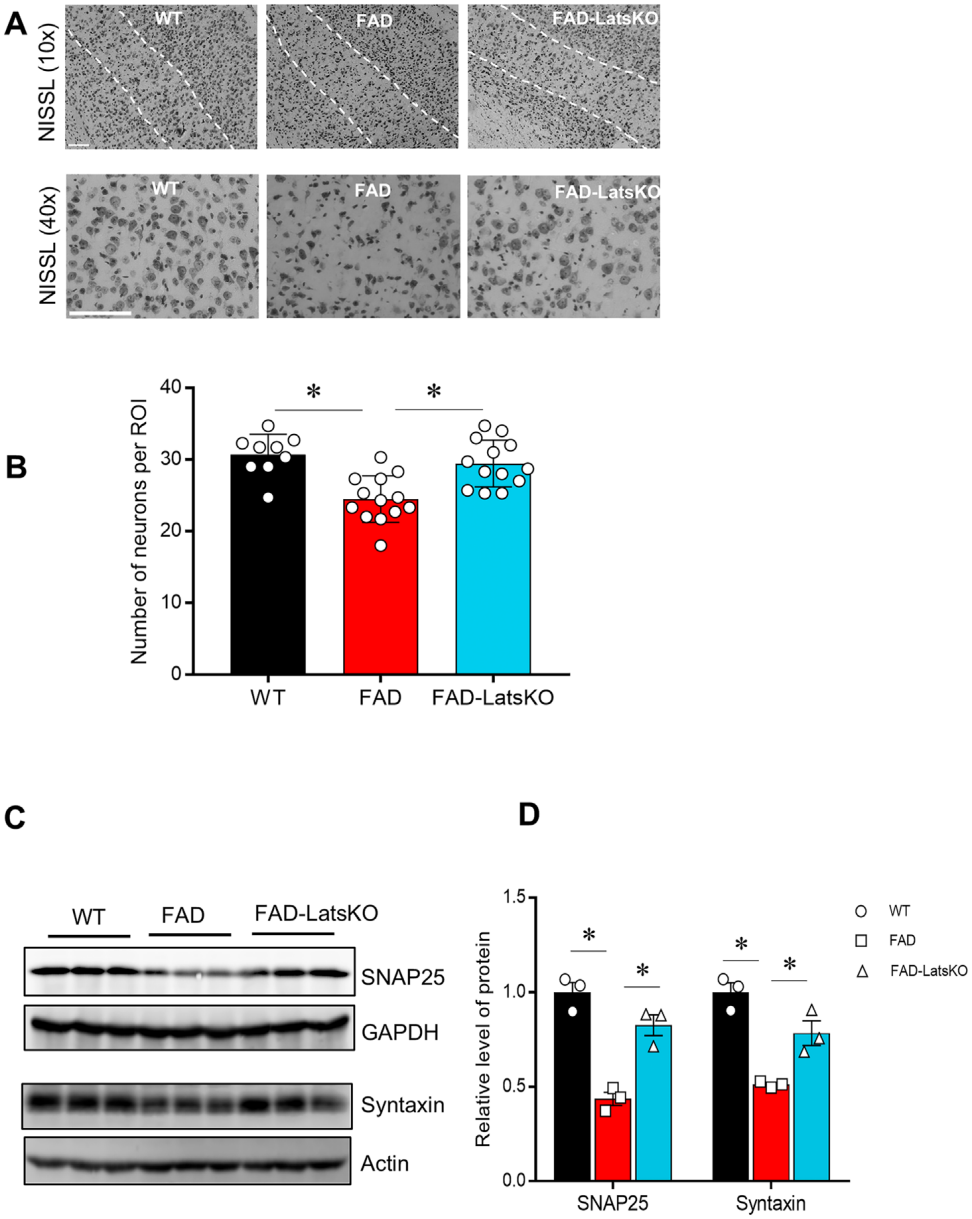
FAD mice with neuronal knockout of Lats1 and Lats2 showed reduced neuronal loss in the cortex. (A) Representative images of Nissl‐stained cortices at 10× and 40× magnification, respectively. The dotted white lines in the upper panels are used to demarcate cortical layer V, which is shown at greater magnification in the lower panels. The scale bars represent 100 μm. (B) Numbers of neurons in cortical layer V region. *p < 0.05. (C, D) Images and quantified results of immunoblotting showing levels of synaptic protein SNAP25 and Syntaxin. Error bars represent SEM. *p < 0.05.

To evaluate the effect of KO of Lats1 and Lats2 on neurodegeneration in 5xFAD mice, we performed Nissl staining on brain sections from cohorts of mice. The region of interest for this assay was layer V of the cortex, as this is one of the few areas where neuronal degeneration and death have been observed in 5xFAD mice (Oakley et al. 2006; Ohno et al. 2007). The results show that FAD mice exhibit clear signs of neuronal loss in cortical layer V compared to the WT group (Figure 4A,B). However, the Nissl‐stained sections from FAD‐LatsKO mice show neuronal density and morphology in cortical layer V similar to that observed in the WT groups, indicating that FAD‐LatsKO mice had ameliorated neuronal death compared to FAD mice in this vulnerable region.

5xFAD mice also exhibited neurodegeneration as exemplified by the loss of synaptic proteins. To determine whether neurodegeneration was ameliorated in FAD‐LatsKO mice, we compared levels of synaptic proteins in cortical tissues between WT, FAD, and FAD‐LatsKO mice. As shown in Figure 4C,D, SNAP25 and Syntaxin, two synaptic marker proteins, were found to be higher in FAD‐LatsKO mice compared to FAD mice, indicating that neurodegeneration was ameliorated by KO of Lats1 and Lats2.

### 2.6 Primary Culture Neurons With KO of Lats1 and Lats2 Showed Increased Resilience Against Ferroptosis

**FIGURE 5:**
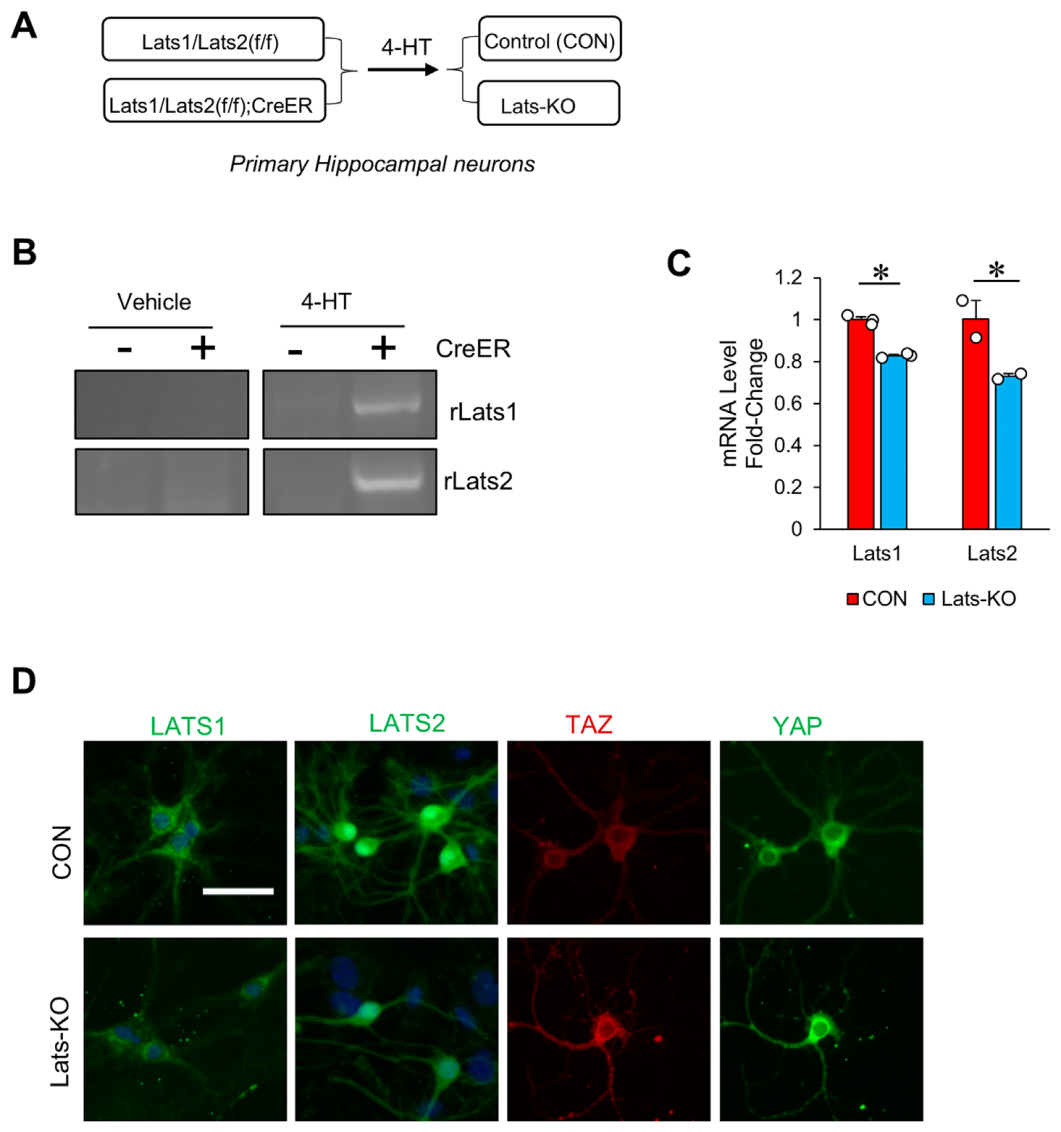
Generation of primary culture hippocampal neurons with knockout of Lats1 and Lats2. (A) Scheme for generating primary culture Control (CON) and LatsKO neurons. (B) PCR gel images showing the detection of rLats1 and rLats2 only in Lats1/Lats2(f/f); Camk2α‐CreER neurons after 4‐HT treatment. (+): Lats1/Lats2(f/f); Camk2α‐CreER or “LatsKO” neurons, (−): Lats1/Lats2(f/f) or “CON”. (C) RT‐qPCR results showing mRNA levels of Lats1 and Lats2 in CON and LatsKO neurons, normalized to GAPDH. Error bars represent SEM. *p < 0.05. (D) Representative images of immunofluorescence staining of LATS1, LATS2, YAP, and TAZ in LatsKO and CON neurons. Scale bar represents 50 μm.

The observation that downstream Hippo signaling inhibition by KO of Lats1 and Lats2 helped to prevent cognitive decline and reduce neurodegeneration in 5xFAD mice prompted us to investigate how KO of Lats1 and Lats2 may be beneficial for the survival of neurons. To that end, primary hippocampal neuronal cultures were generated from P0 to P1 Lats1/Lats2(f/f); Camk2α‐CreER mice and Lats1/Lats2(f/f) mice, respectively (Figure 5A). The Lats1/Lats2(f/f); Camk2α‐CreER and Lats1/Lats2(f/f) primary neuronal cultures were then treated with 4‐hydroxytamoxifen (4‐HT) to generate LatsKO and control (CON) neurons, respectively.

To verify KO of Lats1 and Lats2, PCR testing for the presence of deletion amplicons from rLats1 and rLats2 was performed on DNA isolated from LatsKO and CON neurons. The results show that 4‐HT treatment (1 μM) for 48 h resulted in the generation of rLats1 and rLats2 in LatsKO neurons (i.e., neurons positive for CreER, Figure 5B). As expected, no presence of rLats1 or rLats2 was detected in CON neurons, which are negative for CreER. We also assessed levels of Lats1 and Lats2 mRNA by RT‐qPCR. Consistent with KO of Lats1 and Lats2, RT‐qPCR results showed a significant decrease in mRNA levels of Lats1 and Lats2 in LatsKO neurons compared to CON neurons (Figure 5C). To further confirm KO of Lats1 and Lats2 and downstream Hippo signaling inhibition in LatsKO neurons, we examined the protein levels of LATS1 and LATS2 as well as YAP and TAZ by IF. The IF results indicated that LatsKO neurons exhibited reduced LATS1 and LATS2 proteins compared to CON neurons (Figure 5D). Moreover, the IF results indicated that LatsKO neurons had increased nuclear and cytosolic protein levels of YAP and TAZ (Figure 5D), which is consistent with reduced activity of LATS1 and LATS2, which normally phosphorylate these proteins, thereby tagging them for cytoplasmic retention and/or degradation (Kim and Jho 2014; Pan 2010; Ramos and Camargo 2012).

**FIGURE 6:**
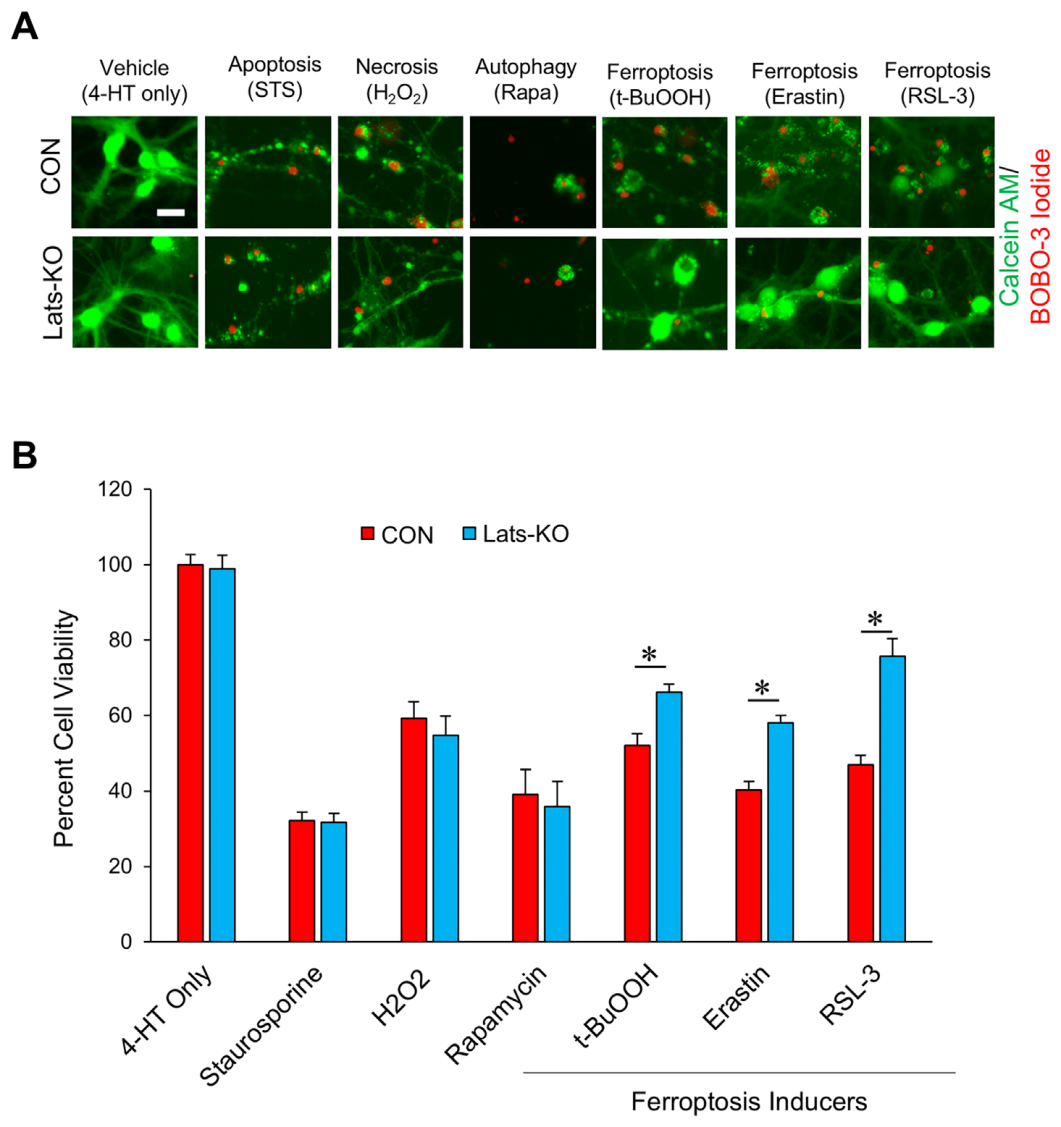
Increased resilience of LatsKO neurons against chemical inducers of ferroptosis. (A) Representative images of CON and LatsKO neurons treated with chemical inducers of apoptosis (staurosporine), necrosis (H_2_O_2_), autophagic cell death (rapamycin), ferroptosis (*t*‐BuOOH, Erastin and RSL‐3), or Vehicle. The red signal is BOBO‐3 Iodide, which is indicative of dead cells. The green signal is calcein‐AM, which is a live cell indicator. Scale bar represents 25 μm. (B) Percentages of cell viability measured as the ratio of living (green) cells to total cells (green and red) for the conditions seen in (A). For each condition, *n* = 10–12 for the number of fields of view analyzed. Error bars represent SEM. *p < 0.05.

To determine how inhibition of Hippo signaling may affect neuronal survival, we assessed cell viabilities of CON and LatsKO neurons following treatment with chemical inducers of apoptosis (staurosporine, STS), necrosis (hydrogen peroxide, H_2_O_2_), or autophagic cell death (rapamycin, Rapa) (Bertrand et al. 1994; Raught et al. 2001; Ali et al. 2013). The representative images of treated CON and LatsKO neurons labeled with live/dead fluorescent dyes are shown in Figure 6A, and the quantified levels of percent cell viability are shown in Figure 6B. According to those results, LatsKO and CON neurons showed similar levels of resistance/vulnerability to apoptosis, necrosis, and autophagic cell death as no differences in cell viabilities were observed between CON and LatsKO neurons after treatment with the chemical inducers of apoptosis, necrosis, or autophagic cell death. We further compared cell viabilities of CON and LatsKO neurons following exposure to chemical inducers of ferroptosis. Neurons were treated with either t‐BuOOH, erastin, or RSL‐3, all of which are specific inducers of ferroptosis that function through separate but related mechanisms (Wang et al. 2016; Wenz et al. 2018; Yang et al. 2014). The results of Figure 6A,B revealed that LatsKO neurons had significantly increased survival compared with CON neurons after treatment with those ferroptosis inducers, indicating that KO of Lats1 and Lats2 increased neurons' resilience against ferroptosis.

### 2.7 Primary Culture Neurons With KO of Lats1 and Lats2 Showed Reduced Lipid Peroxidation

**FIGURE 7:**
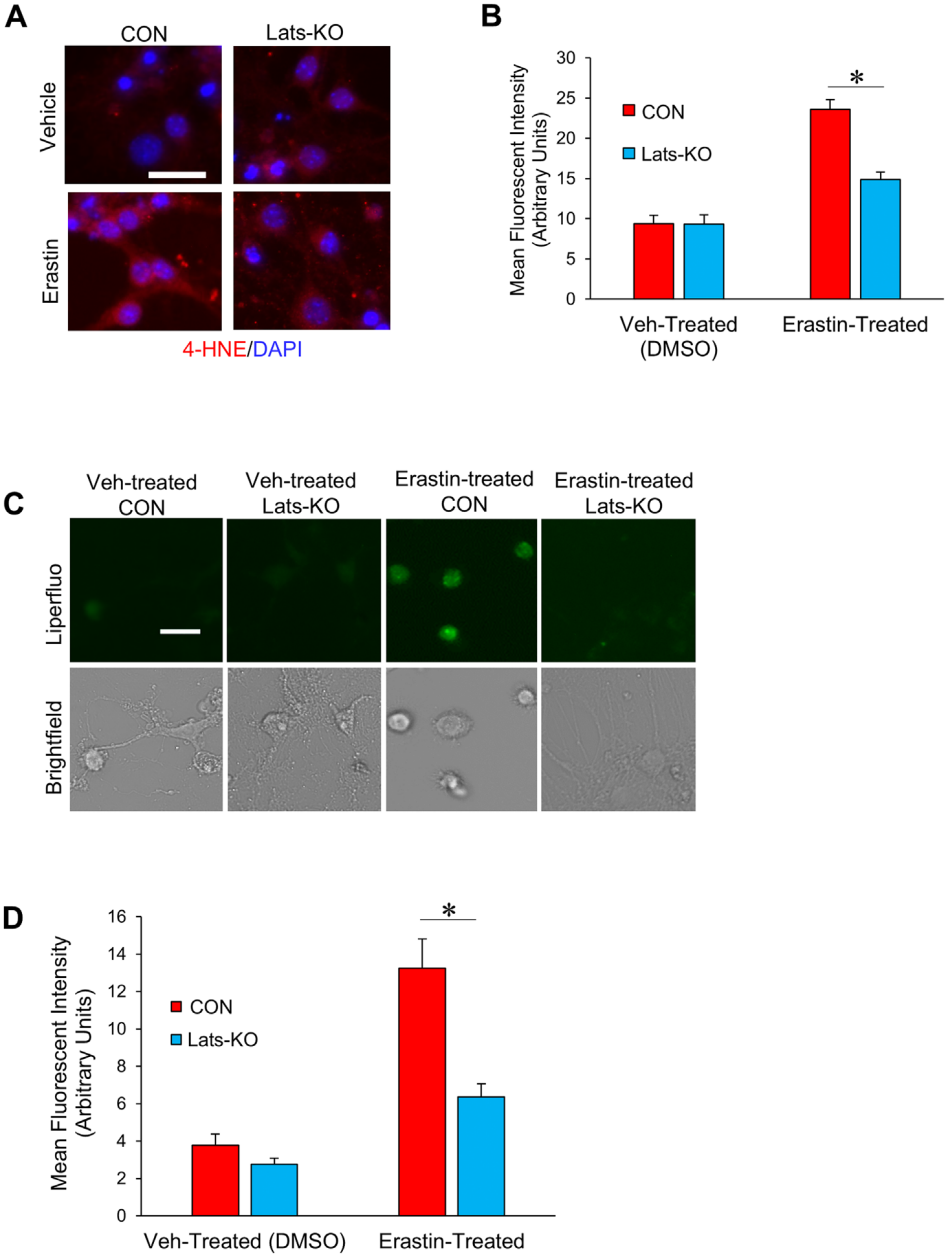
LatsKO neurons exhibited reduced lipid peroxidation. (A) Representative immunofluorescence images from CON and LatsKO neurons treated with erastin (10 μM) or Vehicle (DMSO) and stained with an antibody specific to 4‐HNE protein adducts. (B) Quantified mean fluorescence intensity of 4‐HNE in neurons (*n* = 12–16 cells analyzed per condition). (C) Representative images of CON and LatsKO neurons loaded with Liperfluo, a fluorescent dye indicative of lipid ROS. Scale bars represent 25 μm. (D) Quantified mean fluorescence intensity of Liperfluo in neurons (*n* = 10–12 cells analyzed per condition). Error bars represent SEM. *p < 0.05.

We next wanted to understand why the ablation of Lats1 and Lats2 and downstream Hippo pathway inhibition conferred increased resistance specifically against ferroptosis. To that end, we treated CON and LatsKO neurons with either erastin or vehicle (DMSO) and then fixed and stained these neurons with an antibody specific to 4‐hydroxylnonenal (4‐HNE), a marker of lipid peroxidation as well as ferroptosis (Chen et al. 2022; Feng and Stockwell 2018; Yamada et al. 2020). Representative images of 4‐HNE‐stained neurons are presented in Figure 7A, and the fluorescence intensities for each group were quantified and presented in Figure 7B. Both vehicle‐treated LatsKO and CON neurons had very low levels of 4‐HNE fluorescence, signifying minimal lipid peroxidation. In CON neurons treated with erastin, the level of 4‐HNE fluorescence was significantly elevated. The erastin‐treated LatsKO neurons also had a higher level of 4‐HNE fluorescence compared to vehicle‐treated LatsKO neurons; however, compared to erastin‐treated CON neurons, erastin‐treated LatsKO neurons showed a significantly reduced level of 4‐HNE fluorescence, suggesting that those neurons have decreased lipid peroxidation.

To verify that LatsKO neurons have reduced lipid peroxidation, we also compared the levels of lipid reactive oxygen species (ROS) in CON and LatsKO neurons. After treatment with erastin or vehicle, the neurons were loaded with Liperfluo, a fluorescent dye that detects lipid ROS (Yamanaka et al. 2012). Representative images of Liperfluo fluorescence (green) and brightfield channels for erastin‐ or vehicle‐treated neurons can be seen in Figure 7C, and the quantified levels of Liperfluo fluorescence are presented in Figure 7D. As shown, erastin‐treated CON neurons had a much higher fluorescence intensity than cells treated with vehicle, indicating increased lipid ROS levels caused by erastin treatment (Figure 7D). Significantly, the Liperfluo fluorescence intensity of erastin‐treated LatsKO neurons was much lower than that of erastin‐treated CON neurons, indicating that LatsKO neurons had reduced lipid ROS levels.

### 2.8 FAD‐LatsKO Mice Exhibited Reduced Markers of Ferroptosis and Transcriptomic Enrichment of Metabolic Pathways Associated With Ferroptosis

**FIGURE 8:**
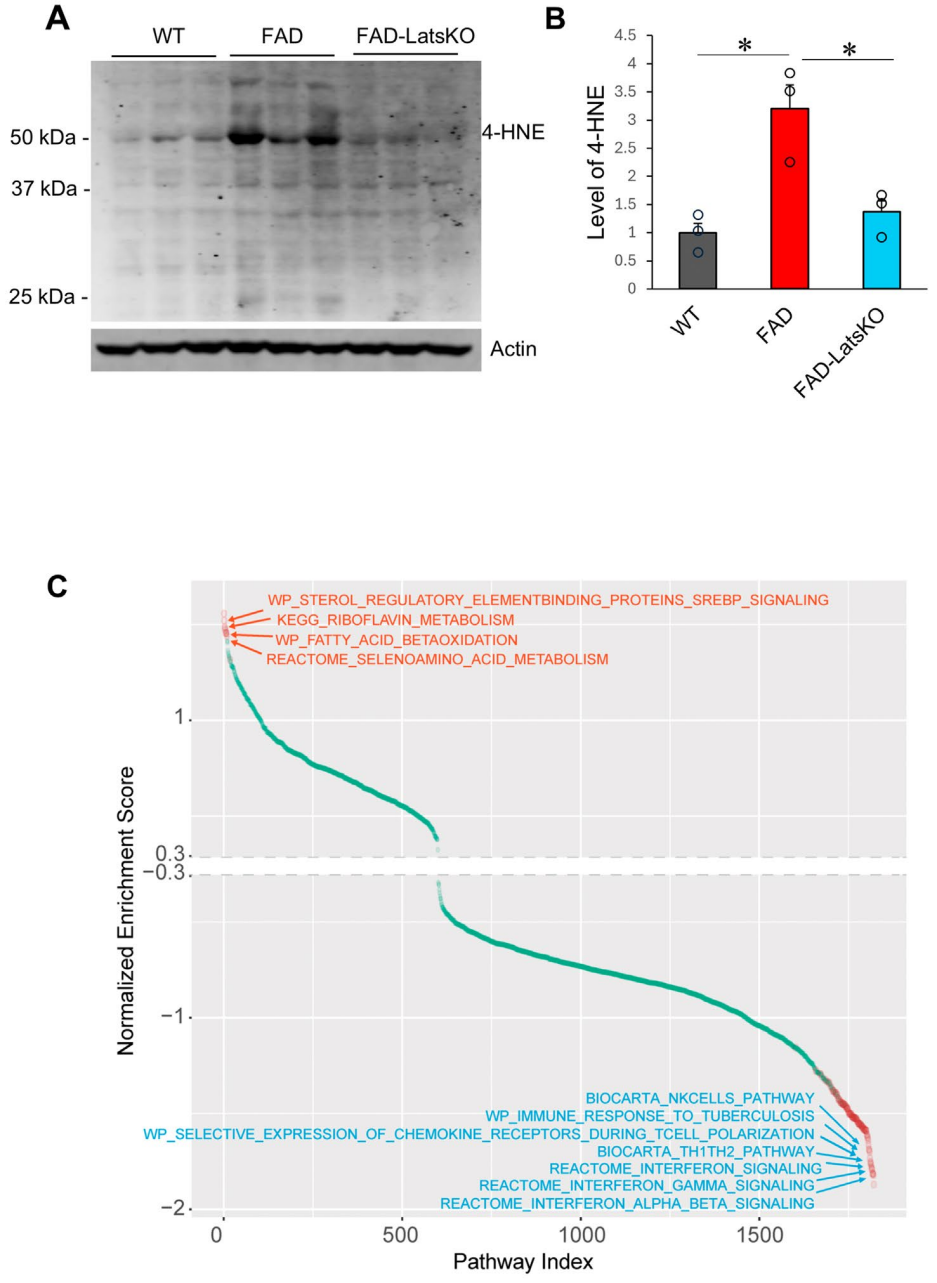
FAD mice with neuronal knockout of Lats1 and Lats2 exhibited reduced markers associated with ferroptosis. (A) Images of immunoblotting results showing 4‐HNE protein in cortices from WT, FAD, and FAD‐LatsKO mice, with Actin as a loading control. (B) Quantification of immunoblotting results from (A) Error bars represent SEM. *n* = 3, *P < 0.05. (C) Up‐ and down‐regulated gene pathways in FAD‐LatsKO mice compared with FAD mice. Significant pathways are shown as red dots (*p* < 0.05, *n* = 4).

We reported previously that ferroptosis is a key mode of neurodegeneration in 5xFAD mice (Chen et al. 2022). The results of increased resilience of LatsKO neurons to ferroptosis prompted us to determine if ferroptosis might be altered in FAD‐LatsKO mice. To that end, we compared 4‐HNE protein adduct levels in the cortices of FAD‐LatsKO and FAD mice using immunoblotting (Figure 8A). As reported before, FAD mice had an increased level of 4‐HNE protein adducts compared to WT mice. Notably, FAD‐LatsKO mice showed a lower level of 4‐HNE protein adducts than FAD mice (Figure 8B).

To determine how KO of Lats1 and Lats2 might affect ferroptosis, we profiled transcriptomes of FAD and FAD‐LatsKO mice. In concordance with previous reports on transcriptomic changes in the 5xFAD model (Forner et al. 2021), FAD mice exhibited upregulated pathways involved in inflammation and downregulated pathways involved in synaptic transmission and signaling in comparison with WT mice. Compared with FAD mice, FAD‐LatsKO mice showed upregulation of pathways predominantly involved in regulation of metabolism (Figure 8C). Ferroptosis is regulated by multiple cellular metabolic pathways (Jiang et al. 2021), in particular, pathways that regulate cellular lipid composition and lipid metabolism (Liang et al. 2022). Interestingly, the SREBP1 signaling pathway, a regulatory pathway of lipogenesis shown to suppress ferroptosis (Yi et al. 2020), is one of the most upregulated pathways in FAD‐LatsKO mice. Another upregulated pathway in FAD‐LatsKO mice is the fatty acid beta‐oxidation pathway, which can affect cellular lipids to affect ferroptosis (Liang et al. 2022). Other upregulated signaling pathways in FAD‐LatsKO mice with ties to ferroptosis regulation include riboflavin metabolism and seleno amino acid metabolism, both of which play important roles in cellular antioxidant defense (Plantone et al. 2021; Olfat et al. 2022; Rahmanto and Davies 2012). The downregulated pathways in FAD‐LatsKO mice are predominately pathways involved in regulation of immune response and cytokine production (Figure 8C, GSEA‐RNAseq in Appendix S1), suggesting that FAD‐LatsKO mice had reduced inflammation, an outcome that is in concordance with ferroptosis suppression (Chen et al. 2021). The reduced neuroinflammation is supported by the reduced astrogliosis and more ramified morphology of microglia in FAD‐LatsKO mice (Figure S3). The upregulated and downregulated genes in FAD‐LatsKO mice are indicated in Figure S4 and Table S1.

## 3 Discussion

The Hippo signaling pathway is an evolutionarily conserved serine/threonine kinase signaling pathway involved in controlling organ size and development (Pan 2010; Harvey et al. 2003; Wu et al. 2003; Halder and Johnson 2011). However, emerging evidence indicates that Hippo signaling may also play important roles in aging (Sladitschek‐Martens et al. 2022) and neurodegenerative diseases including HD, ALS, and AD (Xu et al. 2018; Tanaka et al. 2020; Gogia et al. 2021). In this study, we showed that aged 5xFAD mice had reduced levels of YAP and TAZ positive neurons. Our results thus indicate that there is a hyperactivation of Hippo signaling in neurons afflicted in AD. Despite reports of hyperactivation of Hippo signaling in neurodegenerative diseases, whether the Hippo signaling pathway could be a target for disease ameliorating remains largely unknown. To determine how inhibition of Hippo signaling might affect AD‐related cognitive impairments and pathology, we generated 5xFAD mice with conditional KO of Lats1 and Lats2 in forebrain neurons. Our results indicated that, compared to control 5xFAD mice, 5xFAD mice with neuronal KO of Lats1 and Lats2 mice showed significantly improved cognitive abilities. Moreover, while the overall Aβ burdens were unchanged, 5xFAD mice with neuronal KO of Lats1 and Lats2 mice showed a marked reduction in neurodegeneration compared to control 5xFAD mice. Our results thus indicate that downstream Hippo signaling inhibition in neurons is effective in improving cognition and decreasing neurodegeneration in AD mice.

To investigate how inhibition of Hippo signaling activity confers neural protection in AD mice, we examined the resilience of neurons with KO of Lats1 and Lats2 against chemical inducers of several modalities of cell death. Our results indicated that neurons with KO of Lats1 and Lats2 did not exhibit altered resilience against apoptosis, necrosis, or autophagic cell death compared to control neurons. However, neurons with KO of Lats1 and Lats2 exhibited increased survival when treated with three different inducers of ferroptosis. These results thus indicated that inhibition of Hippo signaling increases neurons' resilience specifically against ferroptosis. Our results further indicated that neurons with KO of Lats1 and Lats2 had reduced 4‐HNE level and decreased lipid ROS. Therefore, KO of Lats1 and Lats2 appears to increase neuronal resilience against ferroptosis by reducing lipid peroxidation. Consistent with data from neurons with KO of Lats1 and Lats2, 5xFAD mice with KO of Lats1 and Lats2 exhibited a lower level of 4‐HNE protein adducts. Ferroptosis was previously shown to be a key mode of neurodegeneration in 5xFAD mice (Chen et al. 2022), the reduced 4‐HNE level in 5xFAD mice with KO of Lats1 and Lats2 therefore suggests that the neural protective effect observed in those mice was mediated by suppression of ferroptotic cell death.

Altered activity of the Hippo signaling pathway activity has been shown to affect cells' vulnerability to cell death mechanisms (Meng et al. 2016; Yang et al. 2019; Wu et al. 2019). Although increased YAP and TAZ activity due to inhibited Hippo signaling was originally reported to induce ferroptosis (Yang et al. 2019; Wu et al. 2019), emerging evidence suggests that the Hippo signaling pathway may regulate cellular sensitivity to ferroptosis in a cell‐type‐dependent manner (Magesh and Cai 2022). Consistent with reports indicating that increased YAP/TAZ activity may confer resistance to ferroptosis in some specific cell types (Gao et al. 2021; Wang et al. 2022; Zhang et al. 2022), we showed that KO of Lats1 and Lats2 in neurons resulted in resistance to ferroptosis. To investigate the underlying causes of KO of Lats1 and Lats2 on resistance to ferroptosis, we compared the transcriptomes of 5xFAD mice with or without KO of Lats1 and Lats2 by bulk RNA sequencing. The results indicate that, compared with control 5xFAD mice, 5xFAD mice with KO of Lats1 and Lats2 had enriched signaling pathways predominantly involved in the regulation of metabolism. Two of the prominently enriched pathways regulate SREBP1 signaling and fatty acid oxidation, both of which have been implicated in the regulation of cells' propensity to undergo ferroptosis through affecting the cellular lipid composition and lipid metabolism. Two other enriched signaling pathways in 5xFAD mice with KO of Lats1 and Lats2 regulate riboflavin metabolism and seleno amino acid metabolism, both of which are associated with defense against ferroptosis. The results of transcriptomic changes thus suggest that inhibition of Hippo signaling altered cellular metabolism to affect cellular lipid composition and anti‐ferroptosis defense to increase resistance to ferroptosis. 5xFAD mice with KO of Lats1 and Lats2 also showed downregulation of signaling pathways involved in the regulation of immune response and cytokine production, suggesting reduced neuroinflammation and altered microglia activity. The reduced neuroinflammation in 5xFAD mice with KO of Lats1 and Lats2 was supported by their reduced astrogliosis and morphological changes of microglia. While reduced neuroinflammation could contribute to the beneficial outcomes such as improved cognition, we believe the reduced neuroinflammation is likely a secondary outcome of reduced neuronal ferroptosis, as KO of Lats1 and Lats2 occurred in neurons in a cell‐type‐specific manner.

Our study provides evidence for the first time on the beneficial effects of downstream Hippo signaling inhibition in neurons in ameliorating AD symptoms and pathology. However, our study was conducted in only one AD model, 5xFAD mice, which lack any form of tau dysregulation or neurofibrillary tangle formation, another hallmark of AD. Future studies should expand to include other AD models to determine the effectiveness of Hippo signaling inhibition in ameliorating AD‐related pathologies. Additionally, in order to validate the beneficial role of Hippo signaling inhibition for the treatment of AD, different approaches for inhibiting Hippo signaling activity, including both genetic and pharmacological means, should be used to verify the results from 5xFAD mice with KO of Lats1 and Lats2. Inhibition of Hippo signaling activity can lead to abnormal growth and cancer (Harvey et al. 2013), so a concern of targeting Hippo signaling for neurodegenerative diseases is whether this approach could inadvertently lead to increased cancer. In this study, we observed no increased proliferation or tumorigenesis in mice months after KO of Lats1 and Lats2. However, more exhaustive examinations and long‐term observation are needed to assess the safety of targeting the Hippo signaling pathway for neurodegenerative diseases. If our observations about the beneficial effects of inhibiting downstream Hippo signaling activity on AD symptoms and pathology and the general safety are confirmed in future studies, the Hippo signaling pathway could provide a new target for AD therapeutic development which could possibly be applied to other neurodegenerative diseases and conditions.

## 4 Methods

### 4.1 Animal Cohorts and Knockout of Lats1 and Lats2

Mice harboring floxed Lats1 and Lats2 alleles were generously provided by Dr. Pei Wang (Liu et al. 2019). These mice were then crossed with 5xFAD mice and Camk2α‐CreER mice as previously described (Chen et al. 2022; Hambright et al. 2017), and back‐crossed as appropriate until 5xFAD; Lats1/Lats2(f/f); Camk2α‐CreER mice were generated. For ablation or knockout (KO) of Lats1 and Lats2 selectively in neurons of the forebrain and hippocampus, mice were injected intraperitoneally (i.p.) with Tamoxifen (TAM) at a daily dose of 60 mg/kg of bodyweight for 4 days (for a total of 240 mg/kg of bodyweight). TAM‐treated mice harboring floxed Lats1 and Lats2 alleles and the Camk2α‐CreER transgene are referred to as LatsKO, whereas TAM‐treated mice without the Camk2α‐CreER transgene (or mice treated with vehicle corn oil) are referred to as CON mice.

CON and LatsKO mice with either the WT or 5xFAD genotype were enrolled in the gender‐balanced experiment cohorts. All animal procedures were reviewed and approved by the Institutional Animal Care and Use Committee (IACUC) of the University of Texas Health Science Center at San Antonio, as well as the IACUC at the Audie Murphy Memorial Veterans Hospital, South Texas Veterans Health Care System.

### 4.2 Tests of Cognition and Behavioral Disinhibition

To evaluate cognitive function of mice, a series of behavioral tests were employed. Behavioral tests were performed on the mice at two time points, first at 5 months of age when 5xFAD mice have mild or no cognitive impairments, then at 9 months of age when 5xFAD mice are expected to have developed profound cognitive impairments (Forner et al. 2021; Jawhar et al. 2012; Schneider et al. 2014; Devi and Ohno 2010).

For the Morris water maze (MWM) test, mice were trained over the course of 5 days of acquisition trials, four trials per day, to locate an obscured underwater platform in a circular tank filled with opaque water. Mice were given a maximum of 60 s to locate the platform. Probe trials were conducted on Day 6, which involved removing the underwater platform and then allowing the mice to swim in the arena for a single 60‐s trial.

For the Y‐maze test, mice were placed in the center of a Y‐shaped maze and allowed to freely explore and move between its three arms for 5 min. At the end of each arm, there was a small illustration, such as a star or square, providing a visual cue for the mice to associate with each arm to help them to remember which arms they had visited recently. Spontaneous alternations, referring to each successful rotation between the three separate arms the mice made without re‐entering the arm they had just been in, were recorded.

For the Open Field test, mice were placed in a large square arena lit by a bright light and surrounded by high walls and allowed to explore freely for 5 min. The proportions of time that the mice spent in the outer edge of the arena adjacent to the walls and corners versus the inner center of the arena were recorded, along with the total distance traveled through the arena.

All tests were video‐recorded using a fixed‐position camera located above the testing apparatus for later review and analysis with the ANY‐Maze video‐tracking software version 7.1 created by Stoelting (Chicago, IL).

### 4.3 PCR Procedures for Genotyping and Detecting Recombinant Alleles of Lats1 and Lats2

Offspring from breeding pairs were genotyped by removing a small portion of tail tissue and processing it using the DirectPCR Tail reagent (Viagen, Los Angeles, CA) following the manufacturer's instructions in order to extract DNA. The DNA was then subjected to polymerase chain reaction (PCR) using specially designed primers meant to detect the presence of wild‐type or floxed alleles for Lats1 and Lats2, as well as primers for detecting the 5xFAD transgenes and the Camk2α‐CreER transgene.

For detecting the presence of the Camk2α‐CreER transgene, the following primer sequences were used: (forward: 5′‐AGC TCG TCA ATC AAG CTG GT‐3′; reverse: 5′‐CAG GTT CTT GCG AAC CTC AT‐3′). For detecting the presence of the floxed alleles of Lats1 and Lats2, the following primers were used: Lats1 (forward: 5′‐TTG TTG CTG CTG TTG TTT CC‐3′; reverse: 5′‐AGA CCT CGT CGC ACA GAA TG‐3′), and Lats2 (forward: 5′‐GCG CAT GCC TTT AAT CCT AGC‐3′; reverse: 5′‐CTG AGC AAC GAC TCC AGG AAC‐3′).

For detecting the deletion amplicons of Lats1 and Lats2 following the KO procedure, DNA was extracted from tissues or cells and subjected to PCR amplification. The following primers were used: rLats1 (forward: 5′‐AGG ATG TAG TGA AGG CGT GTA AC‐3′; reverse: 5′‐AGA CCT CGT CGC ACA GAA TG‐3′), and rLats2 (forward: 5′‐CTA TCG CTA GGC TGT TCC CAC‐3′; reverse: 5′‐CTG AGC AAC GAC TCC AGG AAC‐3′).

### 4.4 Brain Tissue Processing and Staining

The mice were humanely sacrificed and underwent transcardial perfusion with warmed phosphate buffered saline (PBS) followed by 4% paraformaldehyde (PFA). After perfusion, the brains of the mice were collected and postfixed in 4% PFA at 4°C overnight and equilibrated in 30% sucrose in PBS for 1–2 days at 4°C. Brains were then flash‐frozen and stored at −80°C until such time that they were sectioned at either 16 or 30 μm.

For immunofluorescent staining, brain sections were washed in PBS before being blocked for 1 h in PBS solution containing 5% Bovine Serum Albumin (BSA) and 0.3% Triton X‐100. After blocking, the sections were incubated overnight at 4°C with the primary antibody diluted in antibody dilution buffer (PBS with 2.5% BSA and 0.15% Triton X‐100). The following day, the sections were washed in PBS with 0.15% Triton X‐100 and then incubated for 2 h, protected from light, with the fluorophore‐conjugated secondary antibody and DAPI (ThermoFisher, MA). The sections were then washed, mounted on glass slides using Fluoromount‐G Reagent (Southern Biotech, Birmingham, AL) and stored at 4°C protected from light.

For determining YAP and TAZ positive neurons, brain sections were co‐stained with antibodies against YAP and NeuN or antibodies against TAZ and NeuN. images were captured using a Zeiss LSM710 confocal microscope, and QuPath 0.5.1 was used for image analysis (Bankhead et al. 2017). For cell detection, a DAPI intensity threshold of 400 was used. For object classifiers, nuclear mean, sum, standard deviation, max, min, and range were the selected parameters for the NeuN classifier. Cytoplasm mean, standard deviation, min, and nucleus standard deviation, and range were the selected parameters for the YAP and TAZ classifiers. Then the object classifiers were loaded to each image to count neurons positive for NeuN and YAP or TAZ in three different ROIs (Region of Interest), and the average of the three ROIs was recorded for each image.

For Nissl‐staining, sections were stained with 0.1% (w/v) cresyl violet for a duration of 5 min before being dehydrated via graded ethanol rinses and then cleared with xylene and stored at 4°C. Brightfield imaging of Nissl‐stained brain sections was performed using a Zeiss Axio light microscope. For comparing numbers of large pyramidal neurons in cortex layer V, images of cresyl violet stained mouse brain sections were captured using a Nikon N‐Storm super‐resolution microscope system. Image analysis software QuPath 0.5.1 was used to semi‐automatically count large pyramidal neurons in cortex layer V as described by Bankhead, P. et al. (Bankhead et al. 2017). In brief, training images were first selected from each image. Cell detection was performed using optical density sum together with other default settings. The object classifier was trained to identify large pyramidal neurons from other neurons and glial cells. Then the object classifier was loaded to each image to count large pyramidal neurons in three different ROIs (Region of Interest, 180 μm × 180 μm) of layer V. The average count of three ROIs was recorded for each image.

### 4.5 Immunoblotting

Following humane sacrifice, the cortices were dissected out and flash frozen in liquid nitrogen and stored at −80°C. Tissue homogenization and protein extraction was followed as described previously (Chen et al. 2022; Hambright et al. 2017). In brief, cortical tissue samples were manually homogenized in ice‐cold 1× RIPA buffer containing protease and phosphatase inhibitors (EMD Biosciences Inc., San Diego, CA). The homogenates were then centrifuged at 13,000 RPM for 15 min at 4°C, and the resulting supernatants were removed, and protein concentrations were determined using the Pierce BCA protein assay kit (ThermoFisher, MA).

Immunoblotting was conducted as described previously (Chen et al. 2022; Hambright et al. 2017). In brief, SDS‐Page gel electrophoresis was performed using 30–60 μg of protein. The protein was then transferred to either a PVDF or nitrocellulose membrane. Membranes were blocked at room temperature for 1 h in Tris Buffered Saline with 0.1% Tween‐20 (TBST) containing 5% milk, and were then incubated overnight at 4°C with the primary antibody diluted in TBST containing 5% milk. The membranes were then washed with TBST before being incubated for 1 h with the fluorophore‐conjugated secondary antibody at room temperature. After additional washes, membranes were imaged using an Odyssey CLx scanner (LI‐COR, Lincoln, NE) and relative protein levels were calculated by measuring optical density using the NIH ImageJ software (version 1.53t) and normalized using GAPDH as a loading control.

### 4.6 Primary Hippocampal Neuron Culturing Procedures

Mouse hippocampal primary neuronal cultures were generated using a published protocol (Beaudoin 3rd et al. 2012). In brief, P0‐P1 neonatal pups were humanely sacrificed and their hippocampi were carefully removed, processed, and plated on 8‐well Nunc Lab‐Tek II CC2 glass chamber slides (ThermoFisher, MA). Four hours later, the plating media was carefully aspirated from the wells and replaced with neuronal maintenance media containing 2 μM Ara‐C (ThermoFisher, MA) in order to inhibit the growth of non‐neuronal cells. The media was then replaced every 2 days.

After 11 days in culture to allow the neurons to recover from the dissection and plating procedure and to grow neurites and processes, primary hippocampal neurons were treated with 1 μM 4‐hydroxy‐tamoxifen (4‐HT) for 48 h to induce Cre recombination and KO of Lats1/2. Following 4‐HT treatment, Lats1/Lats2(f/f); Camk2α‐CreER neurons were referred to as LatsKO neurons and Lats1/Lats2(f/f) neurons that did not express Camk2α‐CreER were referred to as CON neurons.

For immunostaining of primary cultured neurons, neurons plated on 8‐well chamber slides were fixed in 4% PFA for 15 min before rinsing with PBS. The neurons were then stained as described above. Afterwards, chambers were removed from the slides before being coated in Fluoromount‐G Reagent and a coverslip.

### 4.7 Real‐Time Quantitative PCR

Total RNA was isolated from CON and LATSKO neurons using the PureLink RNA Mini kit (ThermoFisher, MA). Reverse transcription was performed using iScript Reverse Transcription Supermix from Bio‐Rad (Hercules, CA). The mRNA levels of Lats1, Lats2, and GAPDH were quantified with the standard RT‐qPCR protocol for SYBR Green PowerUP (Bio‐Rad, Hercules, CA) and were normalized to GAPDH to control for input RNA. Fold change differences in mRNA levels between CON and LatsKO neurons were calculated using the ΔΔCt method. The primer sequences used for RT‐qPCR are as follows: Lats1 (forward: 5′‐GCG ATG TCT AGC CCA TTC TC‐3′; reverse: 5′‐GGT TGT CCC ACC AAC ATT TC‐3′), Lats2 (forward: 5′‐AGC CTG ACA ACA TAC TCA TCG‐3′; reverse: 5′‐AAT CCA GTG CAG AGG CCA AA‐3′), and GAPDH (forward: 5′‐AGG TCG GTG TGA ACG GAT TTG‐3′; reverse: 5′‐GGG GTC GTT GAT GGC AAC A‐3′).

### 4.8 Cell Viability Assays in Primary Neurons

Survival of primary neurons was measured using an Invitrogen LIVE/DEAD fluorescent cell viability kit (Invitrogen, CA), which includes both a live cell indicator (calcein‐AM, excitation/emission: 494/517 nm) and a dead cell indicator (BOBO‐3 iodide, excitation/emission: 570/602 nm) applied 30 min prior to imaging using an EVOS M7000 live‐cell microscope (ThermoFisher, MA). This assay allowed for the visualization of both the living and dead cells present in a field of view and to calculate the ratio of live cells (calcein‐AM‐positive cells) to total cells (calcein‐AM‐positive cells + BOBO‐3 Iodide‐positive cells) to determine overall cell viability. Because primary neurons do not always distribute evenly across a given well or plate, fields of view for imaging were chosen semi‐randomly in order to ensure that an appropriate number of neurons were visible and to avoid imaging areas largely devoid of cells. All cell counts were determined by manual counting using the NIH ImageJ software (version 1.53t).

To evaluate neuronal resilience against apoptosis, necrosis, and autophagic cell death, CON and LatsKO neurons were treated with staurosporine (STS, 100 nM for 24 h), hydrogen peroxide (H_2_O_2_, 300 μM for 1 h), or rapamycin (Rapa, 5 μM for 24 h), respectively. To test for neuronal resilience against ferroptotic cell death, neurons were treated with erastin (10 μM; 24 h), t‐BuOOH (500 μM; 5 h), and RSL‐3 (1.5 μM; 24 h). Each of these chemical inducers has been shown to effectively induce and promote ferroptosis through separate but related mechanisms. Concentrations and durations used for each chemical inducer were chosen in preliminary study to produce a significant but not insurmountable level of cell death.

### 4.9 Measurement of Lipid Peroxidation and Lipid ROS

Neurons were fixed and stained using an antibody specific to 4‐hydroxynonenal (4‐HNE) protein adducts following treatment with erastin (10 μM; 24 h) or vehicle DMSO. Additionally, Liperfluo was used to measure lipid reactive oxygen species (ROS) in neurons following treatment with 10 μM erastin or vehicle (DMSO) for 24 h. Liperfluo was dissolved in DMSO before being added to live‐cell cultures at a concentration of 5 μmol/L for 30 min, as directed by the manufacturer's instructions.

Fixed neurons were imaged using an LSM 780 Confocal Microscope (Zeiss, Germany). Live‐cell neuronal cultures treated with Liperfluo were imaged using the EVOS M7000 fluorescent microscope (Thermofisher, MA). The mean fluorescence intensity for each condition was determined by measuring the mean gray value, which is the sum of the gray values divided by the number of pixels within the specified region of interest, for individual cells using NIH ImageJ software version 1.53t.

### 4.10 Measurement of Aβ Species

Cortical tissues were homogenized and processed so as to generate both SDS‐soluble and an SDS‐insoluble fraction, as described previously (Chen et al. 2022). Levels of Aβ40 and Aβ42 were then quantified by enzyme‐linked immunosorbent assays (ELISA) using commercial kits (Catalog # KHB3481, Catalog # KHB3441, ThermoFisher, MA). The levels of Aβ40 and Aβ42 in both SDS‐soluble and insoluble fractions were expressed as ng/mg of total brain protein.

### 4.11 Antibodies

For immunostaining performed on fixed tissues or cells, the following antibodies were used: YAP (1:150; Cell Signaling Cat# 4912), TAZ (1:150; Proteintech Cat# 66500‐1‐Ig), LATS1 (1:150; Proteintech Cat# 17049‐1‐AP), LATS2 (1:150; Proteintech Cat# 20276‐1‐AP), 4‐HNE (1:100; Invitrogen Cat# MA5‐27570), Amyloid Beta (1:250; BioLegend Cat# 803015), and Ki‐67 (1:100; Cell Signaling Cat# 9129).

For immunoblotting, the following antibodies were used: TAZ (1:500; Proteintech Cat# 66500‐1‐Ig), pTAZ (1:500; Cell Signaling Cat# 59971), YAP (1:500; Proteintech Cat# 13584‐1‐AP), pYAP (1:1000; Cell Signaling Cat# 4911), LATS1 (1:500; Proteintech Cat# 17049‐1‐AP), LATS2 (1:500; Proteintech Cat# 20276‐1‐AP), GAPDH (1:5000; Sigma Cat# G9545), Procaspase‐3 (1:500; Cell Signaling Cat# 9662), 4‐HNE (1:500; Invitrogen Cat# MA5‐27570). Snap 25 (1:5000, ABCAM, ab5666), and Syntaxin 1 (1:2,000, ABCAM, ab272736).

### 4.12 RNAseq Data Analysis

RNAseq was performed by the Genome Sequencing Facility at UTHSA. The quality of raw RNA‐seq data reads was checked with FastQC (v0.12.1). Low‐quality reads and sequence adapters were removed using TrimGalore (v0.6.10). Kallisto (v0.44.0, mouse genome assembly version GRCm39) was used to process RNAseq data and generate gene counts and TPM. Differentially expressed genes (DEG) were identified using the DESeq2 (Dysken et al. 2014) package (v1.40.2) with default parameters in R (v4.3.2). Genes with a *p*‐value < 0.05 and an absolute value of log2FoldChange ≥ 1 were considered differentially expressed.

Gene set enrichment analysis was implemented using the fGSEA package (v1.28.0) with default parameters in R (v4.3.2). Gene sets from C2 WikiPathways, BioCarta, KEGG, and Reactome were extracted using MSigDBr. Genes were pre‐ranked according to DESeq2 log2 fold change before used as input for GSEA.

### 4.13 Statistical Analyses

All statistical analyses were performed using the GraphPad PRISM software version 8.3.0 (San Diego, CA). Student's *t*‐test was used where specified to determine differences between two groups, whereas two‐way ANOVA was used for analyses involving three or more groups, with Bonferroni's correction being used for tests of multiple comparisons. All data are expressed as the mean ± SEM. *p* < 0.05 was considered significant for all included analyses.

## Author Contributions

The project was initially conceived by Q.R. and was led by Q.R. and R.C.E. for the duration. R.C.E. performed the majority of experiments and statistical analysis. R.N. supported with genotyping of animals, immunoblotting, immunostaining, and imaging analysis; L.C. provided support with Morris Water Maze testing and ELISA assay experiments, as well as dissections and tissue collection and processing; and N.J.D. conducted primary mouse hippocampal neuronal culturing procedures and helped collect data for some live‐cell and fixed immunocytochemistry experiments with these neurons. J.O. provided guidance in designing the cognitive behavioral testing experiments. J.J. and S.Z. analyzed the RNAseq data. The manuscript was written by R.C.E. and Q.R.

## Conflicts of Interest

The authors declare no conflicts of interest.

## Supporting information


**Figures S1–S4:** acel70218‐sup‐0001‐FiguresS1‐S4.docx.

**Table S1:** acel70218‐sup‐0002‐TableS1.zip.

## Data Availability

The data that support the findings of this study are openly available in Texas Data Repository at https://dataverse.tdl.org/.
