# Supplementary material for: Inhibition of Hippo Signaling Through Ablation of Lats1 and Lats2 Protects Against Cognitive Decline in 5xFAD Mice via Increasing Neuronal Resilience Against Ferroptosis
Source: Aging Cell. 2025 Sep 9;24(11):e70218. doi: 10.1111/acel.70218 (PMC12611316; doi:10.1111/acel.70218)
Supplement: Supplementary file 1 — Figures S1–S4: acel70218‐sup‐0001‐FiguresS1‐S4.docx. [file ACEL-24-e70218-s001.zip › acel70218-sup-0001-FiguresS1-S4/Evans_2025_supplementary_rev.docx]

*Title:*

**Inhibition of Hippo signaling activity through ablation of Lats1 and Lats2 protects against cognitive decline in AD mice via increasing neuronal resilience against ferroptosis**

*Authors:* Robert C. Evans, Nawab John Dar, Liuji Chen, Ren Na, Jason O’Connor, Jing Jiang, Siyuan Zheng, and Qitao Ran

**Supplementary Materials**

Supplemental Figure 1. FAD Mice with Knockout of Lats1 and Lats2 Exhibit No Abnormal Growth in Cortices.

Supplemental Figure 2. Behavior Testing Results for FAD-LatsKO Mice and Other Cohorts at 5 Months of Age.

Supplemental Figure 3. Gliosis in FAD and FAD-LatsKO mice.

Supplementary Figure 4. Differentially expressed genes between FAD-LatsKO and FAD mice.

Supplementary Table 1. Up- and Down- regulated genes in FAD-LatsKO mice


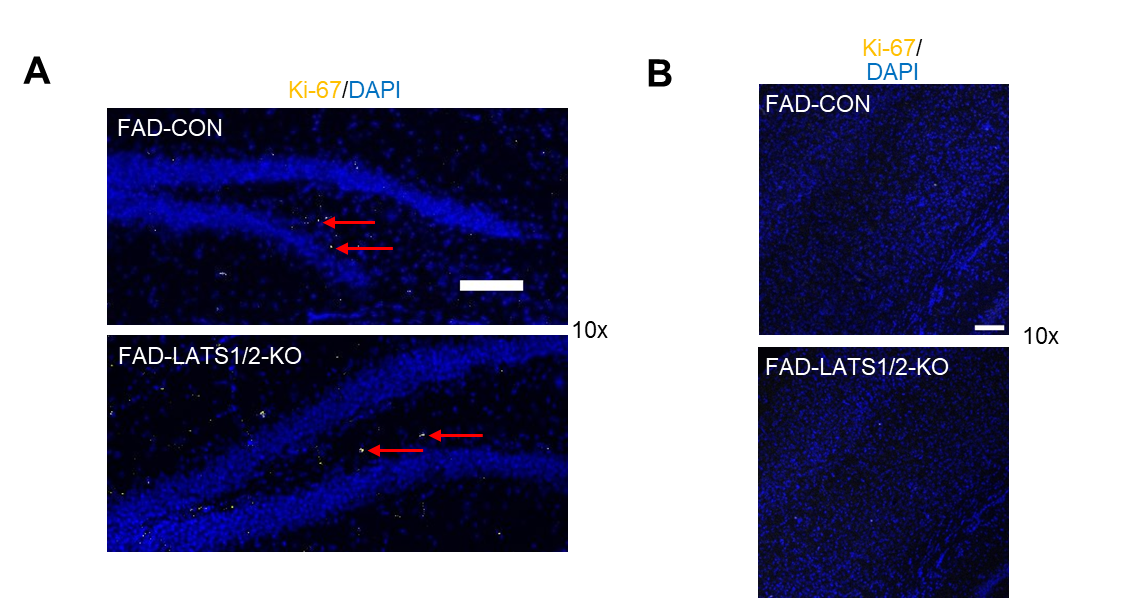


**Supplemental Figure 1. FAD Mice with knockout of Lats1 and Lats2 exhibit no abnormal growth in cortices.**

**A**) Representative images of Ki-67 immuno-stained hilus of the dentate gyrus in brain sections from FAD and FAD-LatsKO mice at 10x magnification. The red arrows point to positive Ki-67 staining likely due to adult neurogenesis. Scale bars represent 100 μm.

**B**) Representative images of Ki-67 immuno-stained cortical regions of FAD and FAD-LatsKO mice at 10x magnification. Scale bars represent 100 μm.


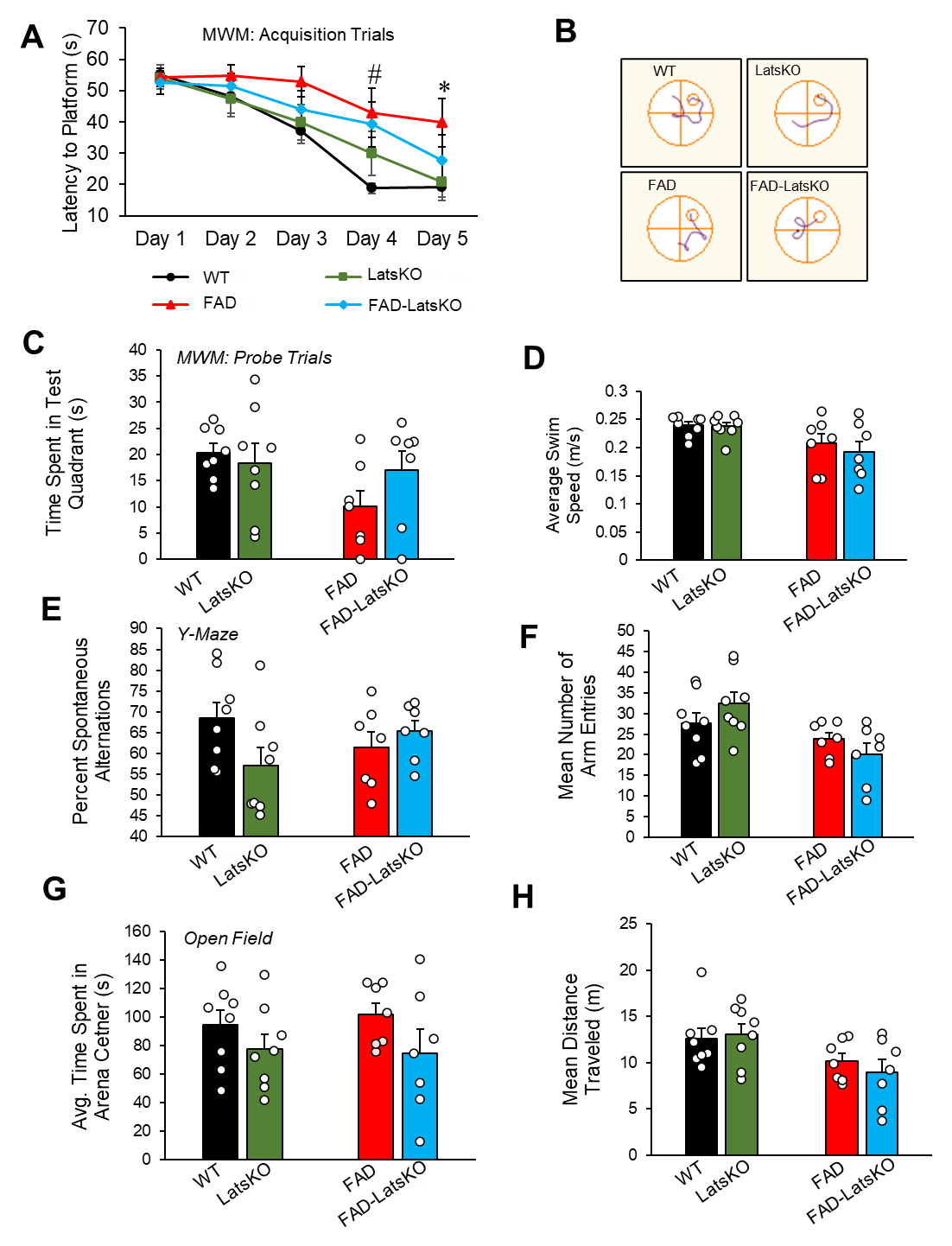


**Supplemental Figure 2. Behavior testing results for FAD-LatsKO mice and other cohorts at 5 months of age.**

**A**) Results of Morris Water Maze (MWM) acquisition trials for the cohorts at 5 months of age. *: *p*<0.05 difference between FAD mice and WT mice, #: *p*<0.05 difference between the WT mice and both FAD mouse groups.

**B**) Representative swim plots of acquisition trials on day 5.

**C**) Results of MWM probe trials on day 6.

**D**) The average swim speed during MWM probe trials.

**E**) Result of Y-Maze Task testing showing spontaneous alternations at 5 months of age.

**F**) The numbers of arm entries performed during the Y-Maze testing.

**G**) Results of Open Field Task testing showing time spent in the center of the arena at 5 months of age.

**H**) Mean distance traveled in the Open Field task. n = 7-8 mice per group. Error bars represent SEM.

Note: None of the differences in B-H are statistically significant.

**
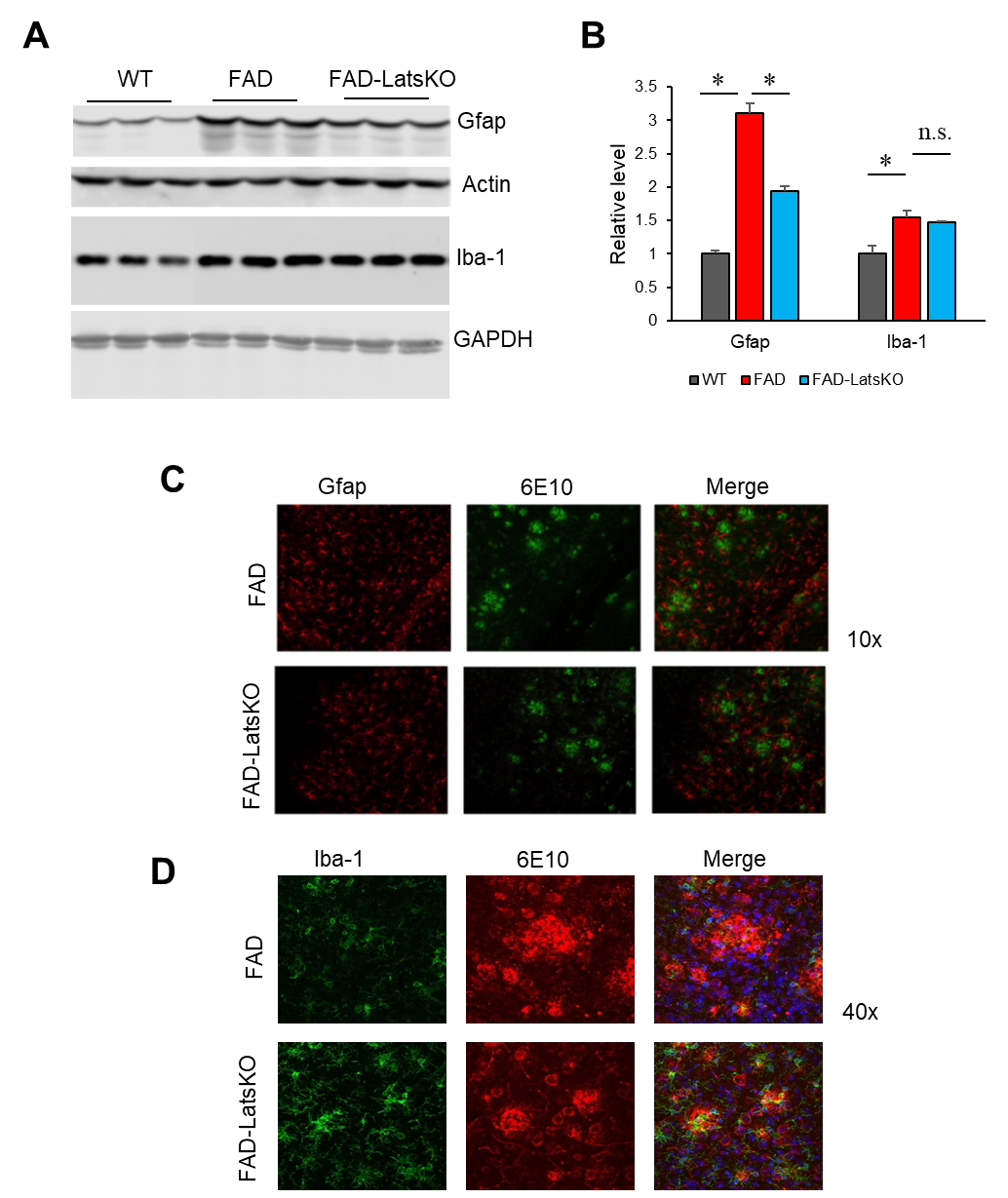
**

**Supplemental Figure 3. Gliosis in FAD and FAD-LatsKO mice.**

**A**) Images of immunoblotting results showing GFAP and Iba-1 proteins in cortices from WT, FAD and FAD-LatsKO mice, with Actin and GAPDH as loading controls.

**B**) Quantification of immunoblotting results from A. Error bars represent SEM. n=3, *: *p*<0.05,

n.s.: nonsignificant statistically

**C-D**) Immunofluorescence images of cortical layer V regions from WT, FAD and FAD-LatsKO mice stained with antibodies against GFAP, Iba-1, and Aβ (6E10).

**Supplementary Figure 4. Differentially expressed genes between FAD-LatsKO and FAD mice.**

Genes upregulated in FAD-LatsKO (log₂ fold change ≥ 1, p < 0.05) are shown in red, while downregulated genes (log₂ fold change ≤ –1, p < 0.05) are shown in blue, not significant genes are shown in black. The top 20 most significant genes (based on p-value) are labeled on the plot. X axis, fold change at log2 scale. Y axis, p value at log10 scale.
