# Supplementary figures and images for: Inhibition of Hippo Signaling Through Ablation of Lats1 and Lats2 Protects Against Cognitive Decline in 5xFAD Mice via Increasing Neuronal Resilience Against Ferroptosis
Source: Aging Cell. 2025 Sep 9;24(11):e70218. doi: 10.1111/acel.70218 (PMC12611316; doi:10.1111/acel.70218)

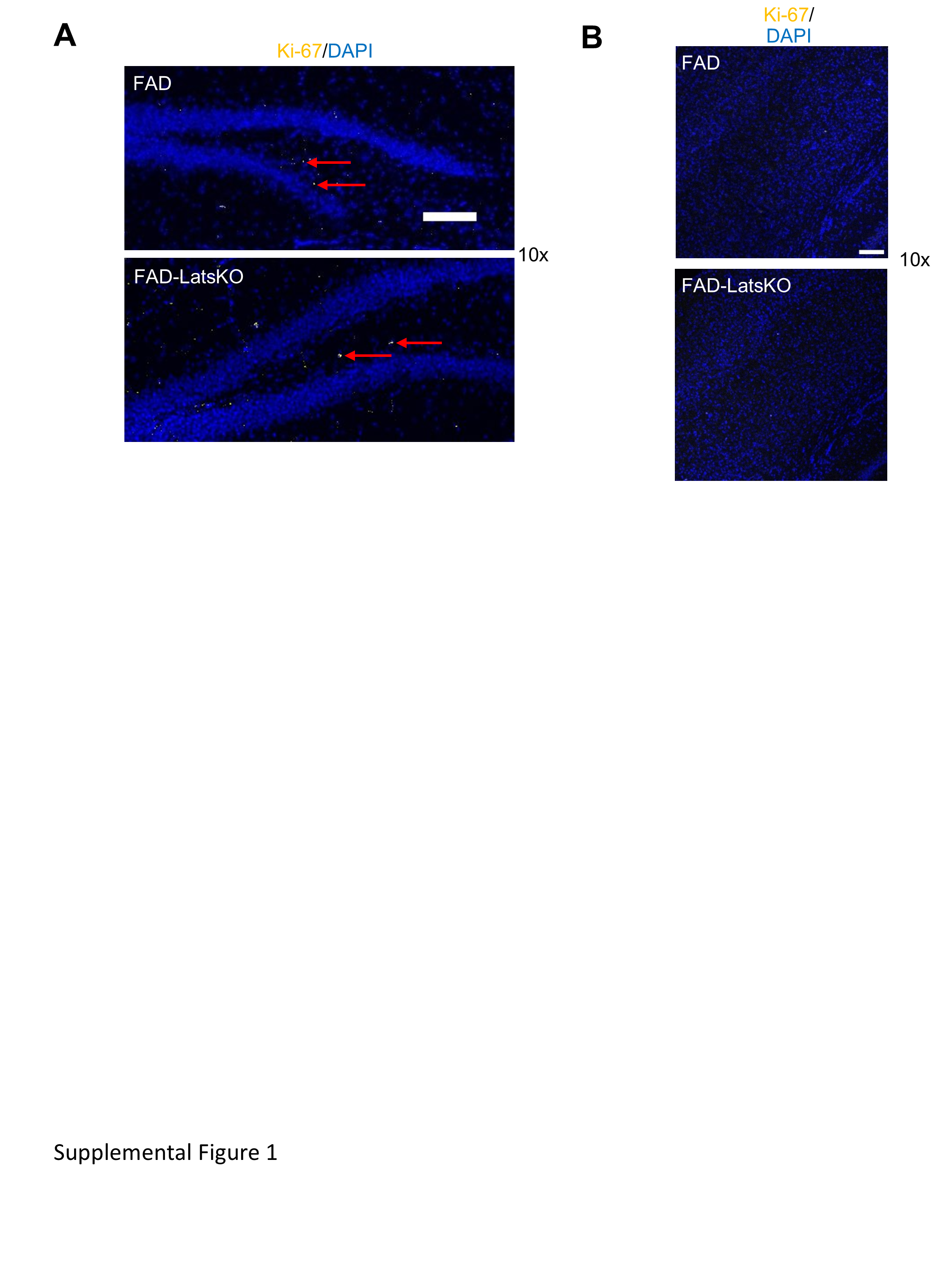

Supplement: Supplementary file 1 — Figures S1–S4: acel70218‐sup‐0001‐FiguresS1‐S4.docx. [file ACEL-24-e70218-s001.zip › acel70218-sup-0001-FiguresS1-S4/Supplementary Figure 1.TIF]

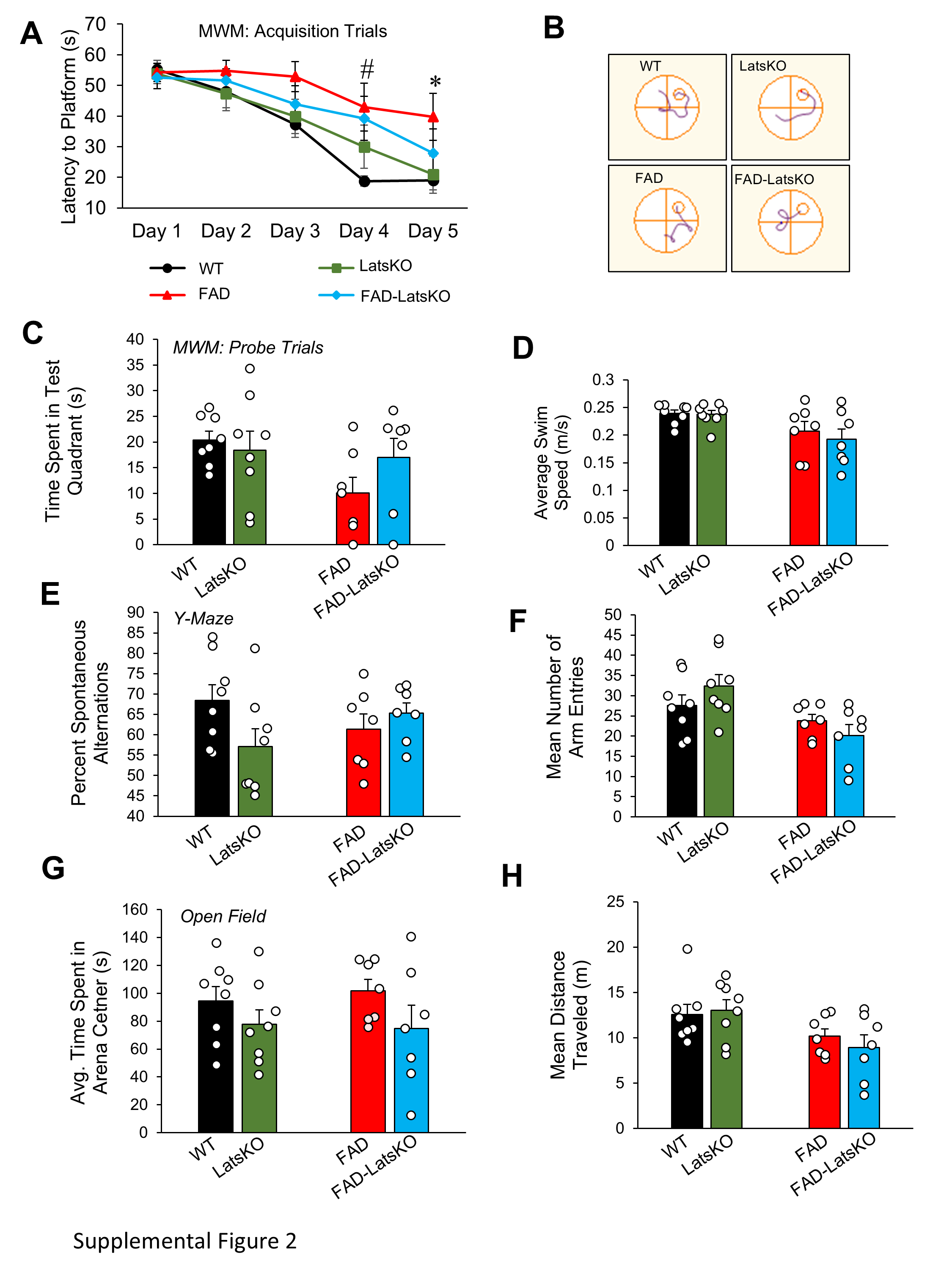

Supplement: Supplementary file 1 — Figures S1–S4: acel70218‐sup‐0001‐FiguresS1‐S4.docx. [file ACEL-24-e70218-s001.zip › acel70218-sup-0001-FiguresS1-S4/Supplementary Figure 2.TIF]

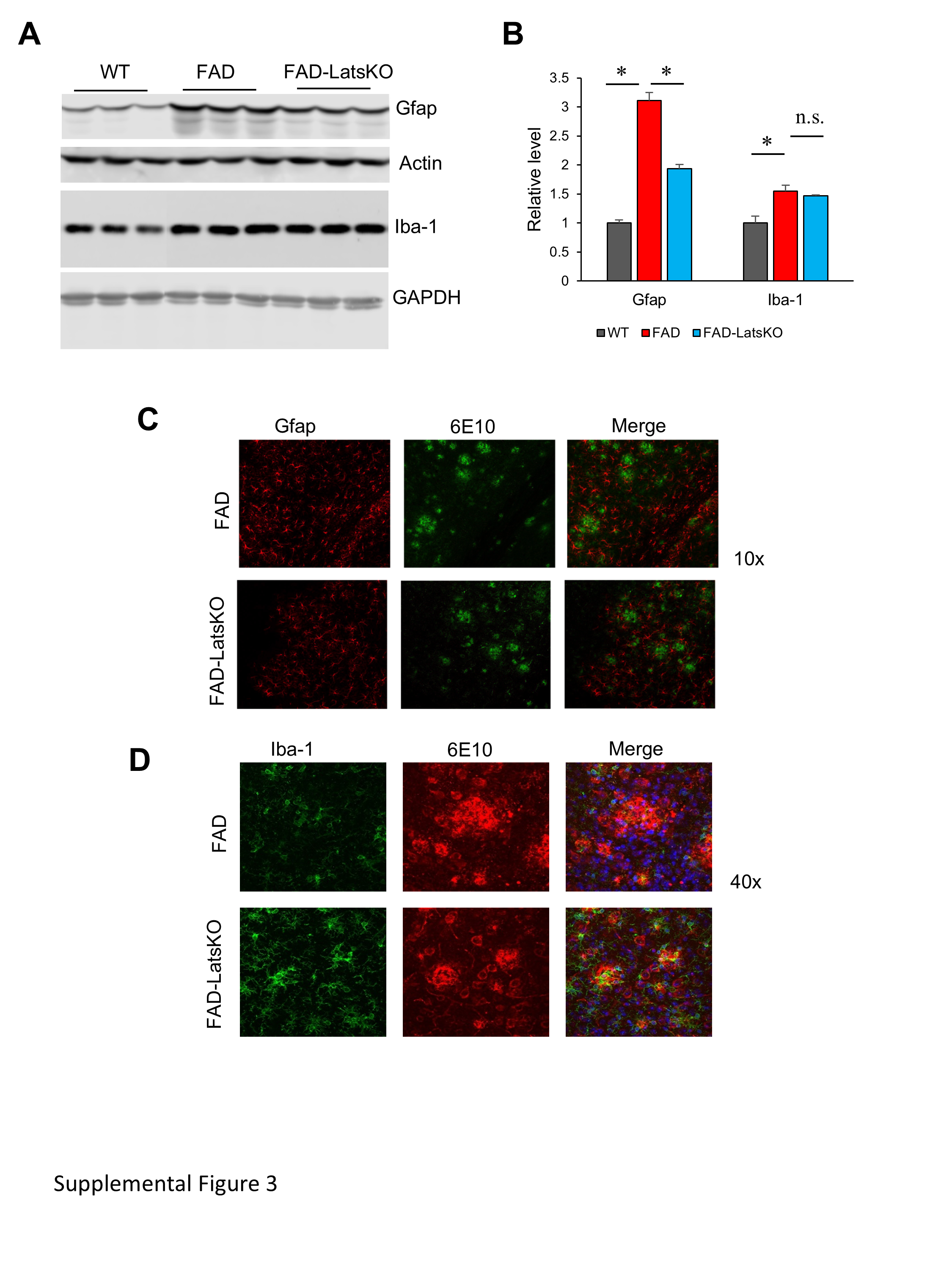

Supplement: Supplementary file 1 — Figures S1–S4: acel70218‐sup‐0001‐FiguresS1‐S4.docx. [file ACEL-24-e70218-s001.zip › acel70218-sup-0001-FiguresS1-S4/Supplementary Figure 3.TIF]

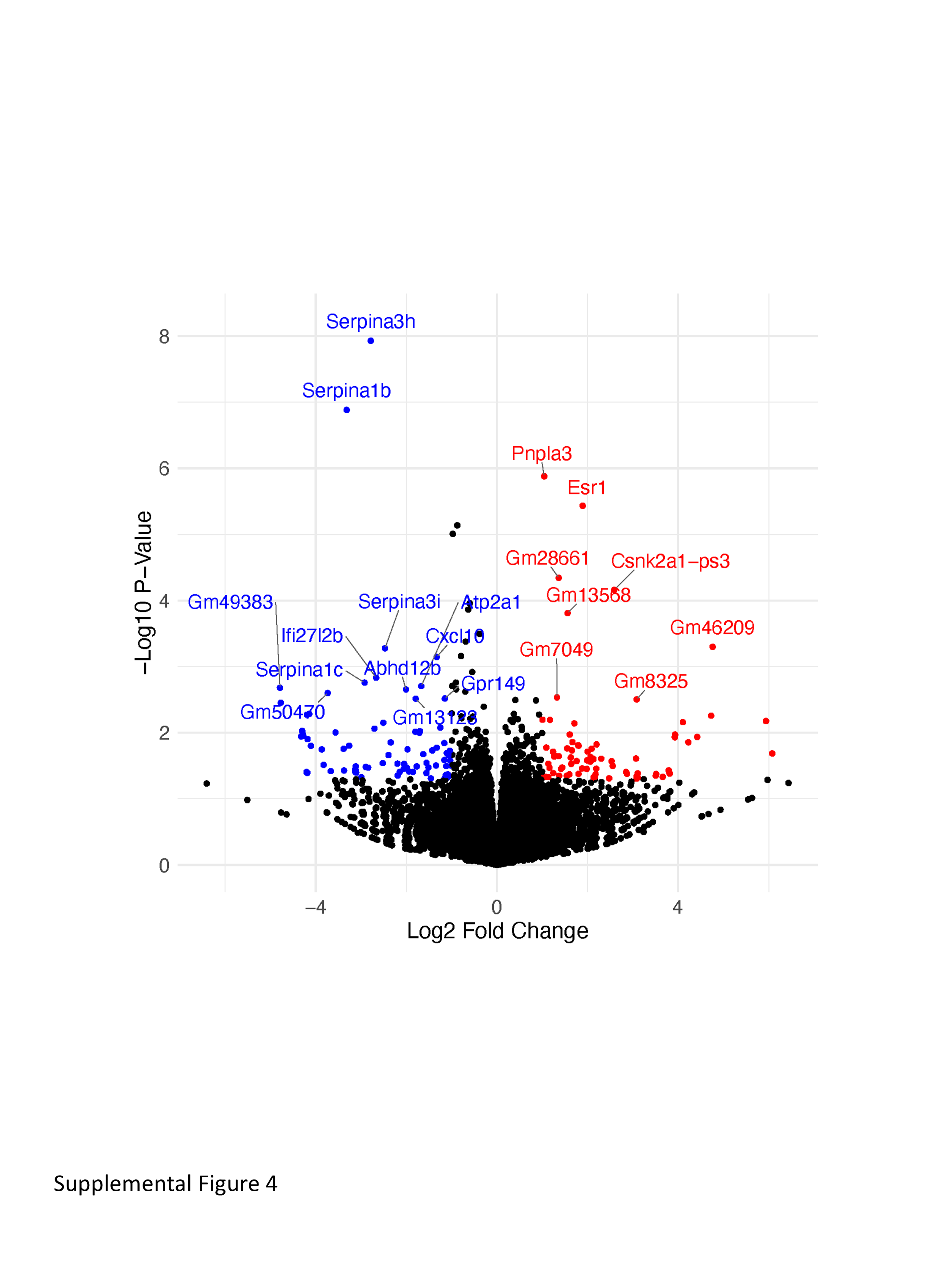

Supplement: Supplementary file 1 — Figures S1–S4: acel70218‐sup‐0001‐FiguresS1‐S4.docx. [file ACEL-24-e70218-s001.zip › acel70218-sup-0001-FiguresS1-S4/Supplementary Figure 4.TIF]
