# Supplementary material for: Inhibition of Hippo Signaling Through Ablation of Lats1 and Lats2 Protects Against Cognitive Decline in 5xFAD Mice via Increasing Neuronal Resilience Against Ferroptosis
Source: Aging Cell. 2025 Sep 9;24(11):e70218. doi: 10.1111/acel.70218 (PMC12611316; doi:10.1111/acel.70218)
Supplement: Supplementary file 2 — Table S1: acel70218‐sup‐0002‐TableS1.zip. [file ACEL-24-e70218-s002.zip › Supplementary-Table-1-FAD-LatsKO_vs_FAD_DEGs.pdf]

FAD-LatsKO vs.FAD; upregulated in FAD-LatsKO, pvalue < 0.05 & log2FoldChange >= 1; down regulated in FAD-LatsKO, pvalue < 0.05 & log2FoldChange <=

Gene symbol	baseMean	log2FoldChange	lfcSE	stat	pvalue	padj	regulate
Serpina3h	31.7901914	-2.785822478	0.488454113	-5.70334531	1.17E-08	0.000466696	down
Serpina1b	13.82824138	-3.318173312	0.628727065	-5.27760534	1.31E-07	0.002599728	down
Pnpla3	129.0042627	1.043514247	0.215734297	4.837034553	1.32E-06	0.017451701	up
Esr1	274.0600479	1.892639863	0.408897991	4.628635765	3.68E-06	0.036556115	up
Gm13456	285.0292268	-0.87550564	0.195203321	-4.48509604	7.29E-06	0.057905493	Not Significant
Rn7sk	63.64489234	-0.975154386	0.220574508	-4.420975	9.83E-06	0.065055657	Not Significant
Gm28661	2196.176994	1.363819343	0.33440833	4.078305535	4.54E-05	0.257453562	up
Csnk2a1-ps3	11.20645967	2.587624844	0.650093679	3.980387637	6.88E-05	0.341658403	up
Cebpd	168.3755963	-0.600636164	0.155311617	-3.86729708	0.000110048	0.485753346	Not Significant
Tcp11l1	661.3884711	-0.635189342	0.166557901	-3.8136248	0.000136943	0.544021042	Not Significant
Gm13568	24.06271863	1.559603301	0.412283782	3.782839318	0.00015505	0.559954267	up
Ifi27	714.8926502	-0.383410552	0.106553949	-3.59827634	0.000320333	0.999977237	Not Significant
Spp1	462.5250413	-0.691198534	0.195886429	-3.52856775	0.000417815	0.999977237	Not Significant
Gm46209	2.439375385	4.761665743	1.368348645	3.479862943	0.00050167	0.999977237	up
Serpina3i	8.309431328	-2.472452289	0.7131375	-3.46700642	0.000526289	0.999977237	down
Gm49708	94.41252538	-0.794076492	0.23404785	-3.3927955	0.000691833	0.999977237	Not Significant
Cxcl10	42.061091	-1.332330157	0.393733336	-3.38383884	0.000714799	0.999977237	down
Hspb1	170.475786	-0.546973032	0.168972168	-3.23705992	0.001207681	0.999977237	Not Significant
Ifi27l2b	5.472122194	-2.668846909	0.838990161	-3.18102289	0.00146756	0.999977237	down
Serpina1c	5.455152622	-2.923833637	0.933823517	-3.13103449	0.001741917	0.999977237	down
Drd2	84.65696221	-0.906588311	0.289559505	-3.1309223	0.001742583	0.999977237	Not Significant
Usp18	64.77754875	-0.988377363	0.319288787	-3.09555926	0.001964421	0.999977237	Not Significant
Atp2a1	17.60503024	-1.671310659	0.540034348	-3.09482289	0.001969305	0.999977237	down
Gm49383	2.589703931	-4.791132998	1.556787042	-3.07757765	0.002086905	0.999977237	down
H2ac19	102.1847464	-0.903246134	0.295077466	-3.06104748	0.002205641	0.999977237	Not Significant
Abhd12b	29.09578384	-2.008423901	0.656316339	-3.06014612	0.00221229	0.999977237	down
Gbp6	152.3321841	-0.697958086	0.229721943	-3.03827347	0.002379379	0.999977237	Not Significant
Gm50470	19.13471528	-3.737223523	1.236589546	-3.0222021	0.002509429	0.999977237	down

Gm7049	16.6801028	1.324827512	0.445154299	2.97610854	0.002919315	0.999977237	up
Gpr149	17.03555885	-1.150491669	0.388047398	-2.96482253	0.003028576	0.999977237	down
Gm13123	12.21225564	-1.7939144	0.605606488	-2.96217831	0.003054708	0.999977237	down
Gm8325	8.963887603	3.083991133	1.043615141	2.95510386	0.003125638	0.999977237	up
Col12a1	290.6400926	0.405084927	0.137409051	2.948022159	0.003198141	0.999977237	Not Significant
Gm43154	35.40485914	0.860657031	0.292355161	2.943874937	0.003241309	0.999977237	Not Significant
Gm8249	2.552395044	-4.772915533	1.635275332	-2.91872288	0.003514685	0.999977237	down
Elmod2	480.8272399	-0.291375675	0.101327655	-2.87557899	0.004032872	0.999977237	Not Significant
Mmp23	20.71439188	-0.997422649	0.356037723	-2.80145216	0.005087318	0.999977237	Not Significant
Gm49384	2.865638579	-4.146451148	1.483593685	-2.79486977	0.005192063	0.999977237	down
Smim36	175.366488	0.373367679	0.133598214	2.794705616	0.005194699	0.999977237	Not Significant
Gm15880	23.21830842	0.928623067	0.333160005	2.787318568	0.00531462	0.999977237	Not Significant
Gm13080	1.709033291	-4.193100026	1.504562542	-2.78692305	0.005321111	0.999977237	down
Or5p58	2.371824908	4.728134673	1.703700358	2.775214932	0.005516524	0.999977237	up
Klhl1	58.77994125	-0.785950542	0.284082763	-2.76662523	0.005663983	0.999977237	Not Significant
Rn7s1	101.9759431	-0.532163024	0.19243737	-2.76538296	0.0056856	0.999977237	Not Significant
Rn7s2	101.9759431	-0.532163024	0.19243737	-2.76538296	0.0056856	0.999977237	Not Significant
Oasl2	223.2461827	-0.783476901	0.285278741	-2.74635572	0.006026139	0.999977237	Not Significant
Lgr5	80.20586411	-0.592079906	0.216134715	-2.73940216	0.006155103	0.999977237	Not Significant
Fhad1	141.6007874	0.392061808	0.143185793	2.738133435	0.0061789	0.999977237	Not Significant
Zfp784	91.59965025	0.461244268	0.16863269	2.735200798	0.006234223	0.999977237	Not Significant
Gm47477	24.43508222	1.004059974	0.367822343	2.72974166	0.006338397	0.999977237	up
Gm15878	13.03233907	1.170449619	0.42908857	2.727757627	0.006376644	0.999977237	up
Tfrc	2472.069722	0.301957525	0.110805077	2.725123546	0.006427743	0.999977237	Not Significant
Gm15725	5.502154489	5.940179858	2.188941792	2.713722165	0.006653194	0.999977237	up
Rpl10a-ps1	642.2419823	0.34653307	0.128279673	2.701387218	0.006905089	0.999977237	Not Significant
Gm12254	1.550515102	4.105555246	1.520169607	2.700721833	0.006918918	0.999977237	up
Gm52989	6.730976778	-2.510186494	0.931611365	-2.69445671	0.007050348	0.999977237	down
Gm49183	8.439808779	1.708484208	0.635912372	2.686666093	0.007216906	0.999977237	up
D030055H07Rik	70.52959207	0.545431067	0.206123931	2.646131695	0.008141811	0.999977237	Not Significant

Slc6a6	4073.606365	0.188519909	0.071389015	2.64074113	0.00827249	0.999977237	Not Significant
B430218F22Rik	13.28879248	-1.249254848	0.473793077	-2.6367098	0.008371442	0.999977237	down
H2ac7	3.765965489	-2.705093827	1.030452179	-2.62515222	0.008661024	0.999977237	down
Cdr1	134.3074745	-0.673458408	0.257053938	-2.61991088	0.008795275	0.999977237	Not Significant
Tmco4	99.17490605	-0.42872475	0.164111994	-2.61239132	0.008991127	0.999977237	Not Significant
Gm32687	139.9792422	0.551240459	0.211292525	2.608897112	0.009083455	0.999977237	Not Significant
Gm6209	44.53564919	-0.625394773	0.240182199	-2.60383482	0.009218717	0.999977237	Not Significant
Gm16568	1.844183603	-4.301626507	1.655419711	-2.59851111	0.009362902	0.999977237	down
Gm37240	9.746873909	-1.699887653	0.655173191	-2.59456229	0.009471145	0.999977237	down
H4c9	22.29078442	-0.914754511	0.353369436	-2.58866336	0.009634924	0.999977237	Not Significant
Oas1g	11.50293946	-1.802814578	0.696690406	-2.58768394	0.009662359	0.999977237	down
Lars2	9921.819094	-0.24787161	0.095864436	-2.5856472	0.009719636	0.999977237	Not Significant
9330162G02Rik	2984.956262	0.236911013	0.091721075	2.582950688	0.009795932	0.999977237	Not Significant
Gm47525	1.957929292	-3.565178635	1.382020272	-2.57968621	0.009889013	0.999977237	down
Irx5	9.157902864	-1.713458923	0.665284561	-2.57552786	0.010008721	0.999977237	down
Gm48795	40.31323311	0.997256343	0.387762232	2.571824334	0.010116421	0.999977237	Not Significant
Bst2	126.6337788	-0.500003815	0.194636358	-2.56891271	0.010201815	0.999977237	Not Significant
Trdv3	1.819809861	-4.285279465	1.67367704	-2.56039807	0.010455233	0.999977237	down
Rpl3	4409.195041	1.606703762	0.628942006	2.554613538	0.010630575	0.999977237	up
Gm43106	2.380353654	3.933037937	1.53991866	2.554055638	0.010647624	0.999977237	up
Adcy4	120.2635764	-0.423813439	0.166191754	-2.55014721	0.010767744	0.999977237	Not Significant
Rtp4	82.96462555	-0.575155242	0.226103164	-2.54377353	0.010966216	0.999977237	Not Significant
Gm45297	47.72044458	0.659798382	0.259886067	2.538798597	0.011123384	0.999977237	Not Significant
H2ac18	68.54418822	0.885563833	0.349069138	2.536929614	0.011182944	0.999977237	Not Significant
Hmgb1-ps2	1.803118452	-4.274067698	1.68554707	-2.53571542	0.011221789	0.999977237	down
Hadhb-ps	1.878894759	-4.325266724	1.708807024	-2.5311616	0.011368546	0.999977237	down
Gm54614	1.922409468	4.420200449	1.750981965	2.524412323	0.011589191	0.999977237	up
Gm10109	2.356091295	3.926253849	1.557387156	2.521051901	0.011700459	0.999977237	up
Amotl1	1117.533173	-0.363070601	0.144054574	-2.52036844	0.011723205	0.999977237	Not Significant
5730424H11Rik	34.48888594	0.658295841	0.261263712	2.519660445	0.011746809	0.999977237	Not Significant

Gm48360	1.694486902	-4.182979189	1.674786675	-2.4976191	0.012503046	0.999977237	down
Gm43652	48.74003328	0.775611822	0.310594968	2.497180904	0.012518508	0.999977237	Not Significant
Gm45627	48.6948533	-0.700240186	0.280480917	-2.49656979	0.012540099	0.999977237	Not Significant
Gucy2e	127.9714543	0.425654897	0.170781536	2.492394121	0.012688515	0.999977237	Not Significant
4930429F24Rik	49.46703156	-0.696121496	0.279880091	-2.48721334	0.012874816	0.999977237	Not Significant
Gm55094	697.8502714	-0.253149537	0.101914664	-2.48393634	0.012993903	0.999977237	Not Significant
Gm55118	697.8502714	-0.253149537	0.101914664	-2.48393634	0.012993903	0.999977237	Not Significant
Gm55936	697.8502714	-0.253149537	0.101914664	-2.48393634	0.012993903	0.999977237	Not Significant
Gm17916	17.83843736	-0.993674432	0.400554123	-2.48074948	0.013110648	0.999977237	Not Significant
Adamtsl2	130.2001228	0.427787132	0.1730942	2.471412288	0.013458056	0.999977237	Not Significant
Mak	130.9147874	-0.387996585	0.157200814	-2.46815888	0.013581003	0.999977237	Not Significant
Gm13344	6.666785523	1.66682906	0.67747684	2.4603484	0.01388022	0.999977237	up
Gm43684	52.30281856	0.800612851	0.325592843	2.458938729	0.01393484	0.999977237	Not Significant
Gm55654	1.681555028	4.224658907	1.718192314	2.45878117	0.013940956	0.999977237	up
Gm49239	3.893016511	-2.346924561	0.955066312	-2.457342	0.013996937	0.999977237	down
Gm18030	34.31605727	0.659786733	0.26851967	2.457126261	0.014005345	0.999977237	Not Significant
Gm48492	18.19893616	-1.163188998	0.474989797	-2.44887154	0.014330455	0.999977237	down
1810049J17Rik	24.8467518	-0.826565589	0.338133806	-2.44449261	0.014505605	0.999977237	Not Significant
Gm14419	123.7017043	0.62935373	0.257678552	2.442398588	0.014590028	0.999977237	Not Significant
Zfp949	747.9251726	0.451860644	0.185242828	2.439288197	0.014716227	0.999977237	Not Significant
Gpr179	43.1696565	-0.774118238	0.318224528	-2.4326165	0.014990168	0.999977237	Not Significant
Gm29253	8.879063686	2.198937299	0.903986927	2.432487941	0.014995491	0.999977237	up
Trim56	423.7398841	-0.249669046	0.102762052	-2.42958408	0.015116158	0.999977237	Not Significant
Gm53013	82.67645928	0.496508347	0.204921952	2.422914397	0.015396554	0.999977237	Not Significant
Gm10635	13.23190783	1.791201572	0.739415106	2.422457367	0.015415934	0.999977237	up
Gm38162	101.1807256	0.485458936	0.200469566	2.421609149	0.01545196	0.999977237	Not Significant
Vrk1	318.8847537	0.280889313	0.116002222	2.421413217	0.015460292	0.999977237	Not Significant
Gm49749	65.87731991	0.531546624	0.21972878	2.419103329	0.01555882	0.999977237	Not Significant
Gm56752	2.738643023	-3.26411045	1.35095371	-2.4161527	0.015685482	0.999977237	down
Gm18212	5.460088501	1.801757169	0.746019017	2.415162521	0.01572819	0.999977237	up

Gm49340	1.615090382	-4.107963442	1.701836135	-2.41384194	0.015785308	0.999977237	down
Gm41442	50.97864482	0.58956345	0.24444132	2.411881302	0.015870447	0.999977237	Not Significant
Pcdhb9	283.7486165	0.428694332	0.177945679	2.409130329	0.015990587	0.999977237	Not Significant
Ddx60	66.36109849	-0.709084201	0.294532728	-2.40748865	0.016062661	0.999977237	Not Significant
Zfp568	114.5651314	-0.449921522	0.187347354	-2.40153657	0.016326377	0.999977237	Not Significant
4931408D14Rik	17.86660897	1.088728434	0.455121476	2.392171083	0.016749033	0.999977237	up
Gm21985	76.68069655	-0.644535169	0.269443432	-2.39209827	0.016752356	0.999977237	Not Significant
Adora2a	135.9928507	-1.329778467	0.556057198	-2.39144187	0.016782341	0.999977237	down
Exoc3l4	45.35667353	-0.64109311	0.26811614	-2.39110227	0.016797873	0.999977237	Not Significant
Stk33	22.4301242	0.815567716	0.341229049	2.390088766	0.016844301	0.999977237	Not Significant
Rbm3	1946.66873	0.395514764	0.165581701	2.38863813	0.01691095	0.999977237	Not Significant
Akr1b8	23.25903988	-0.776885396	0.326188591	-2.38170622	0.017232639	0.999977237	Not Significant
Gm32358	6.68625418	1.550871238	0.651381942	2.380893816	0.017270689	0.999977237	up
Stat1	771.3532967	-0.339291225	0.142578686	-2.37967704	0.017327817	0.999977237	Not Significant
Eif2s3y	1021.312331	0.317505908	0.133448553	2.379238298	0.017348457	0.999977237	Not Significant
Unc93a2	4.512380172	2.090668869	0.879786878	2.376335588	0.017485552	0.999977237	up
Gm24924	4.362545583	-3.385861953	1.424828218	-2.37632994	0.01748582	0.999977237	down
Gm54774	4.901449433	-1.974983284	0.833243522	-2.37023539	0.017776764	0.999977237	down
Gimap7	1.368315427	-3.866893689	1.632447566	-2.36877053	0.017847323	0.999977237	down
A630072M18Rik	135.7594488	0.357610584	0.151331781	2.363089775	0.018123279	0.999977237	Not Significant
Gm19220	2142.305491	0.353323914	0.149639904	2.361161053	0.018217817	0.999977237	Not Significant
4933407I08Rik	6.064054676	1.656437332	0.703275674	2.355317257	0.018506897	0.999977237	up
ENSMUSG000001213[illegible]	16.08234023	-1.428094277	0.606439363	-2.35488387	0.018528495	0.999977237	down
Gm56734	16.16550347	-1.041374883	0.442786565	-2.35186649	0.018679478	0.999977237	down
Me1	1531.290751	-0.229347961	0.097684417	-2.34784594	0.018882329	0.999977237	Not Significant
Mtrfr	233.4558378	-0.288999727	0.123116296	-2.34737185	0.018906376	0.999977237	Not Significant
P4ha3	95.75016963	-0.504933587	0.215528171	-2.34277303	0.019141023	0.999977237	Not Significant
Ap3m1-ps	4.255471691	1.99998233	0.854833008	2.339617577	0.019303494	0.999977237	up
4933413J09Rik	11.01887218	1.238080553	0.529471272	2.338333764	0.019369941	0.999977237	up
Gm44559	196.9187929	0.407304821	0.174417524	2.335228776	0.019531473	0.999977237	Not Significant

P4ha1	1104.359728	-0.327595985	0.140365143	-2.33388418	0.019601787	0.999977237	Not Significant
Pstpip2	260.44679	0.322230736	0.138203151	2.331573009	0.019723165	0.999977237	Not Significant
Gm12751	60.69988625	0.51001119	0.218895845	2.329926317	0.019810046	0.999977237	Not Significant
Gm45591	80.96332971	-0.435475369	0.187569107	-2.3216796	0.020250194	0.999977237	Not Significant
Spata21	11.48858372	-1.105238784	0.476233944	-2.3207896	0.020298202	0.999977237	down
Gm13304	6.05759902	6.078058798	2.624109179	2.316237009	0.020545332	0.999977237	up
Coro1c	1572.13107	0.200058147	0.08639766	2.315550537	0.020582822	0.999977237	Not Significant
Smyd1	54.12584701	-0.624856935	0.270005586	-2.31423706	0.020654722	0.999977237	Not Significant
BB031773	5.680448019	-1.627945907	0.705663532	-2.30697185	0.021056389	0.999977237	down
AI838599	40.29173026	0.569329229	0.247007199	2.304909455	0.021171645	0.999977237	Not Significant
Nlrc5	93.13694998	-0.823285255	0.357374854	-2.30370225	0.021239363	0.999977237	Not Significant
9930120I10Rik	13.85684669	-0.985372775	0.428602152	-2.29903833	0.021502763	0.999977237	Not Significant
Gm16121	18.98265035	0.796660432	0.346534312	2.298936655	0.021508537	0.999977237	Not Significant
Map3k14	201.7008868	-0.318792544	0.13887464	-2.29554182	0.021702093	0.999977237	Not Significant
Gm4951	23.55850844	-1.071020173	0.466586496	-2.29543757	0.02170806	0.999977237	down
Gm47840	8.71778896	1.263749417	0.550558975	2.295393363	0.021710591	0.999977237	up
Ifit1	121.3675172	-0.653398438	0.284693057	-2.2950979	0.021727514	0.999977237	Not Significant
Gm49518	3.152735269	-2.394800354	1.044056865	-2.29374514	0.021805143	0.999977237	down
Vwc2	238.9105321	-0.251301253	0.109981258	-2.28494617	0.022315984	0.999977237	Not Significant
Lcat	565.5268218	-0.352866433	0.154562238	-2.28300546	0.022430047	0.999977237	Not Significant
Gm57152	8.18861051	1.364416757	0.598430183	2.279993213	0.022608091	0.999977237	up
Fam205c	191.5748101	0.525879537	0.230970596	2.276824605	0.022796702	0.999977237	Not Significant
Gm57287	8.73929409	1.263450642	0.555276014	2.275356058	0.022884579	0.999977237	up
Gm11613	327.9376881	0.332583364	0.146179507	2.275170926	0.022895678	0.999977237	Not Significant
Ctps2	1027.732083	0.243879805	0.107312087	2.272621967	0.023048971	0.999977237	Not Significant
Gm15382	4.511079338	2.067087037	0.910893523	2.269296011	0.023250331	0.999977237	up
Fgl2	100.1179825	-0.538026181	0.237179552	-2.26843409	0.023302762	0.999977237	Not Significant
Nod1	218.6526707	-0.313631431	0.138313951	-2.26753288	0.023357693	0.999977237	Not Significant
Mir6236	3707.884046	-0.284450532	0.12554014	-2.26581341	0.02346281	0.999977237	Not Significant
5830462I19Rik	29.61980649	0.708946157	0.312911371	2.26564524	0.023473113	0.999977237	Not Significant

Gm42715	393.9549243	0.237840319	0.105047448	2.264122764	0.023566565	0.999977237	Not Significant
Gm15912	116.5398699	0.354343902	0.156551404	2.263434843	0.023608897	0.999977237	Not Significant
Gm10128	11.39609361	1.631091	0.72087247	2.262662353	0.023656511	0.999977237	up
Scn9a	124.1708805	-0.430319958	0.190224009	-2.26217479	0.023686606	0.999977237	Not Significant
Gm26760	30.89240186	-0.848647263	0.375745048	-2.25857205	0.023910018	0.999977237	Not Significant
Adgrb2	11205.72999	0.157808853	0.069876266	2.258404194	0.023920471	0.999977237	Not Significant
Nt5c1a	106.5967136	-0.45929008	0.203504364	-2.25690532	0.024013992	0.999977237	Not Significant
Vav3	172.2228867	-0.344722951	0.152853803	-2.25524616	0.024117882	0.999977237	Not Significant
Rbm28	1294.407016	-0.215154658	0.09543296	-2.25451099	0.02416404	0.999977237	Not Significant
Purb	14731.58594	0.151506821	0.067334115	2.250075174	0.024444174	0.999977237	Not Significant
Gm28177	2.287824812	3.06956629	1.365089756	2.248618654	0.024536769	0.999977237	up
Inpp5f	2654.676146	-0.151375552	0.067338059	-2.247994	0.024576572	0.999977237	Not Significant
Gm57226	2.896363214	2.300953119	1.023926829	2.247185106	0.0246282	0.999977237	up
Gm11847	25.05150866	0.832390208	0.370457159	2.246927042	0.02464469	0.999977237	Not Significant
Gm48505	3.283294651	2.127789912	0.947741727	2.245115787	0.0247607	0.999977237	up
Gm12840	60.38853528	-0.510888385	0.227794811	-2.2427569	0.024912495	0.999977237	Not Significant
Gm18029	3.270509624	2.130249672	0.951232375	2.239462962	0.025125809	0.999977237	up
Gm43860	51.59455683	-0.506853787	0.226507085	-2.23769507	0.025240947	0.999977237	Not Significant
AA474408	2927.800551	-0.255954044	0.114431339	-2.23674778	0.025302829	0.999977237	Not Significant
Zfp775	185.1170269	0.317443869	0.142083769	2.234202192	0.025469772	0.999977237	Not Significant
Gm43473	14.50317343	0.967416562	0.433505126	2.231615046	0.025640416	0.999977237	Not Significant
C1qtnf2	25.13020171	-0.715016569	0.320501685	-2.2309292	0.025685819	0.999977237	Not Significant
Pafah1b3	167.3074107	-0.364224551	0.163320755	-2.23011797	0.025739612	0.999977237	Not Significant
Gm49961	8.643582614	-1.161693439	0.521299099	-2.22845856	0.025849952	0.999977237	down
ENSMUSG0000012159	17.76782365	0.931353059	0.41831326	2.226448805	0.025984134	0.999977237	Not Significant
Rn18s-rs5	50307.5389	-0.395503055	0.177653929	-2.2262556	0.025997066	0.999977237	Not Significant
Gm44186	3.589064461	1.989258518	0.894043116	2.225014075	0.026080293	0.999977237	up
Oas1a	51.12601612	-0.614920572	0.276548505	-2.22355413	0.026178457	0.999977237	Not Significant
Gm43300	105.4089007	0.426458967	0.191831458	2.223091931	0.026209602	0.999977237	Not Significant
Sorbs1	2713.015965	-0.16562214	0.074521229	-2.22248267	0.026250704	0.999977237	Not Significant

Ifi44l	32.89898219	-1.035333439	0.466107963	-2.22123096	0.026335322	0.999977237	down
Gm13323	30.45425272	0.643784695	0.289867015	2.220965688	0.026353285	0.999977237	Not Significant
Helz2	223.1594542	-0.307514947	0.138612474	-2.21852289	0.0265192	0.999977237	Not Significant
Tmtc3	411.6494024	-0.218448671	0.098503627	-2.21767135	0.026577249	0.999977237	Not Significant
Tesmin	4.258604419	1.760694247	0.794966434	2.214803257	0.026773572	0.999977237	up
Myom1	62.24104419	-0.464418774	0.20982754	-2.21333565	0.026874514	0.999977237	Not Significant
Gm42814	3.226955003	2.53076285	1.14378864	2.21261408	0.026924263	0.999977237	up
H4c14	37.99063716	-0.598125209	0.270990426	-2.20718207	0.027301342	0.999977237	Not Significant
Gssos1	3.805836824	2.059686253	0.933811302	2.205677151	0.027406613	0.999977237	up
Gsr	839.4491952	0.208960045	0.09496848	2.200309458	0.027784947	0.999977237	Not Significant
Srbd1	264.3645106	-0.237183708	0.107873588	-2.198719	0.027897909	0.999977237	Not Significant
Rasal1	2165.936377	0.202623983	0.092212238	2.197365423	0.027994359	0.999977237	Not Significant
Gcn1	3136.313129	-0.245031408	0.111779869	-2.19208887	0.02837309	0.999977237	Not Significant
Crlf1	47.43052738	0.59939177	0.273476611	2.191747835	0.02839772	0.999977237	Not Significant
Ighg2b	14.07466951	-1.555719884	0.710598761	-2.18930847	0.028574427	0.999977237	down
Mt1	3432.071044	-0.205604132	0.093971609	-2.18793882	0.028674059	0.999977237	Not Significant
Serpina1a	2.532045828	-2.522390455	1.154144323	-2.18550696	0.028851696	0.999977237	down
Gm26793	47.51276055	0.685787688	0.313989527	2.184110073	0.028954161	0.999977237	Not Significant
Gm42509	3.47807242	-2.196738575	1.007741219	-2.17986377	0.029267561	0.999977237	down
Iqschfp	95.13135243	-0.439832492	0.201772613	-2.17984238	0.029269147	0.999977237	Not Significant
Trmo	49.78949095	0.504209328	0.231322219	2.179683952	0.029280897	0.999977237	Not Significant
Hddc3	302.6630272	0.55593534	0.255152312	2.178837167	0.029343767	0.999977237	Not Significant
Gm38220	11.73623225	1.13392932	0.520513659	2.178481388	0.029370216	0.999977237	up
S1pr2	82.54451401	-0.386887297	0.177675831	-2.17748973	0.029444047	0.999977237	Not Significant
Trac	19.93385159	-0.858036876	0.394206336	-2.17661868	0.02950903	0.999977237	Not Significant
Gm47027	4.496549914	1.639091341	0.753313893	2.175841115	0.029567143	0.999977237	up
ENSMUSG000001213	3.947275801	-2.050980004	0.942736085	-2.17556115	0.029588091	0.999977237	down
A330023F24Rik	2440.911257	-0.161065228	0.074118582	-2.17307488	0.029774682	0.999977237	Not Significant
1110019D14Rik	37.24353822	-0.544914674	0.251500196	-2.16665706	0.030261013	0.999977237	Not Significant
Lyve1	20.09407635	-0.972943895	0.449307639	-2.16542923	0.030354829	0.999977237	Not Significant

Gm20386	1.323013747	-3.82761853	1.770301489	-2.16212806	0.030608306	0.999977237	down
Rgs9	280.3708217	-0.446665494	0.206641798	-2.16154475	0.030653284	0.999977237	Not Significant
Gm4742	7.304987839	-1.346352405	0.625263477	-2.15325611	0.031298556	0.999977237	down
Chat	18.15951427	-1.00583925	0.467667868	-2.15075552	0.031495501	0.999977237	down
Akap12	1080.314507	-0.184414644	0.08577767	-2.14991435	0.03156199	0.999977237	Not Significant
Gm48427	2.53896748	2.555484527	1.18895564	2.149352289	0.031606484	0.999977237	up
Irx1	27.43610676	-1.12159271	0.523021337	-2.14444924	0.031996908	0.999977237	down
Ms4a6c	38.82640013	-0.544460751	0.254188129	-2.14195978	0.032196718	0.999977237	Not Significant
Ntrk1	3.964844436	-1.771338177	0.827427648	-2.14077712	0.032292016	0.999977237	down
Mmrn1	1.473277598	-3.118409184	1.457260991	-2.13991125	0.03236194	0.999977237	down
Gm2004	165.1024303	-0.592646379	0.277075448	-2.13893502	0.032440931	0.999977237	Not Significant
3110039M20Rik	84.33889532	0.488251571	0.228742084	2.134506964	0.032801304	0.999977237	Not Significant
BC018473	16.00220835	0.934573514	0.438158294	2.132958628	0.03292812	0.999977237	Not Significant
Gm49391	3.220827198	-2.896993583	1.358418499	-2.1326223	0.032955722	0.999977237	down
Psrc1	277.7959078	0.312095624	0.146409921	2.131656254	0.033035116	0.999977237	Not Significant
Naa16	299.7239895	-0.280958421	0.13184064	-2.13104564	0.033085382	0.999977237	Not Significant
Rimbp2	4393.03025	0.177297256	0.083197351	2.131044494	0.033085477	0.999977237	Not Significant
Tmprss7	46.81555259	0.501120304	0.235197978	2.130631849	0.033119484	0.999977237	Not Significant
Gm48271	829.5182694	-0.19232851	0.090275986	-2.1304504	0.033134447	0.999977237	Not Significant
Tigd5	144.9924157	-0.306649211	0.144109836	-2.12788537	0.033346589	0.999977237	Not Significant
BC028471	5.426830914	-1.514166592	0.712033948	-2.12653708	0.033458565	0.999977237	down
Naa11	6.34411002	1.440493679	0.677837044	2.125132717	0.03357554	0.999977237	up
Gm12573	3.75895684	-1.991264172	0.937651319	-2.12367234	0.033697552	0.999977237	down
1700112J05Rik	2.068377376	-2.845551781	1.339964348	-2.12360261	0.033703387	0.999977237	down
4930431P19Rik	92.60572341	0.380119499	0.1790423	2.123070916	0.03374791	0.999977237	Not Significant
Aldh1a1	736.8621311	-0.453310451	0.213631434	-2.12192767	0.033843812	0.999977237	Not Significant
Inmt	15.16787903	1.16003379	0.546961993	2.120867272	0.033932974	0.999977237	up
Mon1a	465.2913152	-0.198325641	0.093516558	-2.12075429	0.033942485	0.999977237	Not Significant
Gm43498	31.16461186	0.658605268	0.310595097	2.120462541	0.033967057	0.999977237	Not Significant
Nts	34.58999203	-0.937952425	0.442974393	-2.11739649	0.034226212	0.999977237	Not Significant

Mx1	29.9002465	-0.905906657	0.42844753	-2.11439347	0.034481675	0.999977237	Not Significant
Gm30340	17.79017093	0.76864675	0.363596186	2.114012134	0.034514231	0.999977237	Not Significant
Or8b41	3.980994626	1.877736512	0.888931605	2.112352066	0.034656263	0.999977237	up
Tubd1	158.8608204	-0.424770002	0.201173351	-2.11146258	0.034732571	0.999977237	Not Significant
Sstr3	548.2782359	0.276628326	0.131060747	2.110687859	0.03479915	0.999977237	Not Significant
Gm37980	3.383576417	2.190737073	1.038346713	2.109831952	0.034872833	0.999977237	up
Slc4a1	10.87220838	-2.056163049	0.975314432	-2.10820529	0.035013236	0.999977237	down
Asb4	26.94885042	-0.714579973	0.339120336	-2.10715754	0.035103926	0.999977237	Not Significant
Gm47547	35.11965655	-0.663953851	0.31519632	-2.10647716	0.035162925	0.999977237	Not Significant
Gm16195	6.253684608	1.415135577	0.671816788	2.106430804	0.035166948	0.999977237	up
Gm43802	5.451590892	1.981418762	0.940663965	2.106404451	0.035169235	0.999977237	up
Plk3	379.9808657	-0.251807833	0.119548469	-2.10632419	0.035176201	0.999977237	Not Significant
Gm20371	38.54550124	0.632582256	0.300361926	2.106066724	0.035198557	0.999977237	Not Significant
Vmn1r179	23.34864258	0.670108805	0.318656001	2.102922282	0.035472564	0.999977237	Not Significant
1500002C15Rik	70.75048693	0.396323676	0.188649342	2.100848442	0.035654273	0.999977237	Not Significant
Ttc38	245.7766709	0.264359208	0.12589972	2.099760089	0.035749951	0.999977237	Not Significant
Gm2824	227.7465853	0.269926677	0.128628196	2.098503172	0.03586072	0.999977237	Not Significant
Ddx11	52.0095945	-0.469574238	0.223815927	-2.09803763	0.035901821	0.999977237	Not Significant
Lats2	861.5253406	-0.228967564	0.109213354	-2.09651617	0.036036427	0.999977237	Not Significant
Mir145a	1.481928689	-3.130039886	1.49600928	-2.09225967	0.036415293	0.999977237	down
Gm42927	775.9385766	0.324981703	0.155364919	2.091731554	0.036462536	0.999977237	Not Significant
Gm12481	109.93705	-0.424851271	0.203356882	-2.08919053	0.036690575	0.999977237	Not Significant
Zfhx3	296.5488102	-0.360241977	0.172640981	-2.0866539	0.036919431	0.999977237	Not Significant
Trim30d	50.64467238	-0.670073228	0.321434098	-2.08463642	0.037102316	0.999977237	Not Significant
Gm54754	1.237934275	3.793342798	1.820195216	2.084030748	0.03715737	0.999977237	up
Gm8893	1.720671192	-3.376347269	1.620981665	-2.08290281	0.037260083	0.999977237	down
Gm7653	1.449522495	-3.114385815	1.497073708	-2.08031562	0.037496593	0.999977237	down
Fcgr1	161.8004724	-0.338117528	0.162575613	-2.07975551	0.037547963	0.999977237	Not Significant
Gm26703	248.8203359	0.258664771	0.124482927	2.077913641	0.037717314	0.999977237	Not Significant
Srrm4os	108.5625868	0.394258451	0.18975417	2.077732738	0.037733982	0.999977237	Not Significant

Gm47832	1.182015661	-3.669915003	1.769968216	-2.07343554	0.038131764	0.999977237	down
Gm32200	74.82255044	0.416687747	0.20109123	2.072132865	0.038253052	0.999977237	Not Significant
Gm27004	25.86575075	0.643422614	0.310570848	2.07174182	0.038289525	0.999977237	Not Significant
Gm42577	60.42501694	0.426159383	0.205834015	2.070403102	0.038414612	0.999977237	Not Significant
Gm14062	3.058588818	-1.942423765	0.938913949	-2.06879849	0.038565	0.999977237	down
Stat2	882.6441772	-0.228340814	0.110380273	-2.06867412	0.038576678	0.999977237	Not Significant
Slc39a4	18.40814416	0.995474061	0.481501405	2.067437499	0.038692948	0.999977237	Not Significant
Rps27rt	31.62591812	-2.143620384	1.038256843	-2.064634	0.038957643	0.999977237	down
Foxb1	12.30162009	-1.855316499	0.89925267	-2.06317597	0.039095912	0.999977237	down
Gm43777	2.000908243	2.851248033	1.382999234	2.061641079	0.03924192	0.999977237	up
Gm21983	1.724255707	-4.2053334	2.040433084	-2.0610004	0.039303002	0.999977237	down
Anxa2	280.4841185	-0.378886911	0.183901529	-2.06027058	0.03937268	0.999977237	Not Significant
Mir3473d	1.4629677	-3.125065494	1.517769243	-2.05898591	0.039495588	0.999977237	down
Prss35	83.44759975	0.366864355	0.178208333	2.058626259	0.039530055	0.999977237	Not Significant
Klhl13	613.720188	-0.277166934	0.134661076	-2.05825574	0.03956559	0.999977237	Not Significant
Adamts15	199.8728917	-0.344727102	0.167586996	-2.05700388	0.039685852	0.999977237	Not Significant
Arid4b	1815.000529	0.138369265	0.067278635	2.056659814	0.03971896	0.999977237	Not Significant
Gm10874	1.248288027	3.80297991	1.849128621	2.056633523	0.039721491	0.999977237	up
Gm45133	366.4134836	-0.206082072	0.100286756	-2.0549281	0.03988595	0.999977237	Not Significant
Gm14018	13.52179449	0.961481289	0.468399938	2.052693031	0.040102358	0.999977237	Not Significant
Gm16295	2.396968511	-3.110456116	1.515436885	-2.05251446	0.040119691	0.999977237	down
Gm31087	20.95882495	-0.796936603	0.388777232	-2.0498541	0.040378671	0.999977237	Not Significant
Gm45193	1.700135075	-4.186711871	2.042874622	-2.04942184	0.040420883	0.999977237	down
Gm49674	19.26209353	0.848924034	0.414649713	2.047328162	0.040625875	0.999977237	Not Significant
H4c18	7.579742944	-1.557697705	0.761249714	-2.04623749	0.04073301	0.999977237	down
4933408N05Rik	7.020152629	1.243059348	0.607584226	2.045904575	0.04076576	0.999977237	up
Cfap92	55.80890973	0.475982737	0.232979899	2.043020613	0.041050398	0.999977237	Not Significant
Tac1	239.3884669	-0.452898023	0.22175453	-2.04233944	0.041117872	0.999977237	Not Significant
Irgm1	375.5983346	-0.329028482	0.161189288	-2.04125526	0.041225462	0.999977237	Not Significant
A430019L02Rik	1.403427815	3.10735813	1.52231847	2.041201096	0.041230843	0.999977237	up

Coro7	1503.604259	0.158325774	0.077601951	2.040229299	0.041327493	0.999977237	Not Significant
Gm18407	1.260095841	3.814762884	1.869890117	2.04010003	0.041340364	0.999977237	up
Gm44508	15.18192222	0.960064276	0.470962642	2.038514716	0.041498485	0.999977237	Not Significant
Gm4793	60.20745333	0.479175241	0.235072069	2.038418443	0.041508103	0.999977237	Not Significant
Gm11770	3.82725785	1.801752733	0.885103668	2.03564034	0.041786484	0.999977237	up
Gm12960	2.019813318	2.8700948	1.410397837	2.034954057	0.041855496	0.999977237	up
Gm45553	26.43086648	0.604358749	0.29718504	2.033610942	0.041990838	0.999977237	Not Significant
Gxylt2	87.20365992	-0.412891722	0.203130114	-2.03264653	0.042088247	0.999977237	Not Significant
Tex19.1	1.026188989	3.512696539	1.72891163	2.031738625	0.042180123	0.999977237	up
Rnf38	1349.909217	0.146068801	0.07189402	2.031723938	0.042181611	0.999977237	Not Significant
BC026513	27.39134467	0.697537185	0.343388382	2.031336006	0.042220921	0.999977237	Not Significant
Wrn	424.2145422	0.204831115	0.100872103	2.030602205	0.042295364	0.999977237	Not Significant
4930431F10Rik	3.320295924	2.175671138	1.072492736	2.028611538	0.042497872	0.999977237	up
Edar	4.402662285	1.599529234	0.78858415	2.028355799	0.042523948	0.999977237	up
1700058P15Rik	8.792908448	-1.087988713	0.536657479	-2.02734287	0.04262736	0.999977237	down
Rap1gap2	6288.441525	0.165800829	0.082094038	2.019645185	0.043420205	0.999977237	Not Significant
Lexis1	9.69766095	-1.129014039	0.559269966	-2.01872818	0.04351548	0.999977237	down
Ifit3b	191.7626542	-0.531110246	0.263112853	-2.01856443	0.043532512	0.999977237	Not Significant
Gm39283	13.40641555	-0.9430148	0.467249405	-2.01822579	0.043567751	0.999977237	Not Significant
Utp18	534.8179328	-0.18416338	0.09127717	-2.01762806	0.043630012	0.999977237	Not Significant
Ap4b1	283.5461428	0.226151513	0.112182482	2.015925376	0.04380778	0.999977237	Not Significant
Gm42890	128.6997994	0.453840673	0.225130694	2.015898697	0.04381057	0.999977237	Not Significant
Mfsd2a	407.0736453	-0.260839641	0.129661058	-2.01170378	0.044251172	0.999977237	Not Significant
Kat6b	1797.219473	0.173932782	0.086511695	2.010511788	0.04437705	0.999977237	Not Significant
Gm43109	1.020368286	3.506735921	1.744349641	2.010340037	0.044395212	0.999977237	up
Gm2814	2.802302407	-2.193022508	1.09122848	-2.00968225	0.04446483	0.999977237	down
B930025P03Rik	6.537611701	1.371909839	0.68285845	2.009069141	0.044529802	0.999977237	up
Gm14022	24.4259839	0.626020246	0.311632519	2.00884121	0.044553976	0.999977237	Not Significant
Pax5	6.176798833	1.543490787	0.76865164	2.00804982	0.044637998	0.999977237	up
Lrguk	122.9977304	-0.360057818	0.179382238	-2.00720998	0.044727309	0.999977237	Not Significant

Rabl2	570.9296926	0.21574979	0.107562891	2.005801333	0.044877448	0.999977237	Not Significant
Hsph1	6003.724344	-0.27802596	0.138674592	-2.00488032	0.044975843	0.999977237	Not Significant
Zbtb21	539.3614816	-0.175112555	0.087522758	-2.00076596	0.045417617	0.999977237	Not Significant
Gm44792	49.85782066	-0.63567757	0.317876081	-1.99976535	0.045525608	0.999977237	Not Significant
Slc1a5	89.93177194	-0.345211145	0.172633826	-1.99967268	0.04553562	0.999977237	Not Significant
Csf2rb2	52.76244192	0.566791046	0.283526914	1.999073167	0.045600438	0.999977237	Not Significant
Gm43481	146.4856022	0.398702413	0.199465007	1.998858939	0.045623619	0.999977237	Not Significant
Khdc1a	1.371380574	3.080962249	1.541386714	1.998824967	0.045627295	0.999977237	up
Gm6313	13.80569503	0.860335503	0.430469216	1.998599368	0.045651719	0.999977237	Not Significant
Tbc1d24	2838.552181	0.151582539	0.075854205	1.998340612	0.045679745	0.999977237	Not Significant
Tgm1	38.92449158	-0.817539142	0.409121101	-1.99828153	0.045686146	0.999977237	Not Significant
Oas3	15.45052861	-0.947029546	0.47416125	-1.99727318	0.045795515	0.999977237	Not Significant
Suv39h1	591.7043941	0.186118273	0.093203391	1.996904518	0.045835556	0.999977237	Not Significant
Fcgr4	14.59822238	-0.848065179	0.425073276	-1.9951035	0.046031593	0.999977237	Not Significant
Mt2	2132.231788	-0.274480726	0.137756022	-1.99251344	0.046314752	0.999977237	Not Significant
Gm14040	1.132240201	3.660511777	1.838148992	1.9914119	0.046435622	0.999977237	up
Dennd2c	42.30594394	0.485010841	0.243588448	1.991107726	0.046469045	0.999977237	Not Significant
Cd6	71.33897408	0.441503196	0.221777078	1.990752159	0.046508141	0.999977237	Not Significant
Gm39323	7.86737712	-1.082839514	0.544143254	-1.98998978	0.046592061	0.999977237	down
Gsg1	9.474450482	1.078197731	0.54186133	1.989803796	0.046612553	0.999977237	up
A730011C13Rik	35.9811202	0.547444919	0.275222407	1.989100108	0.046690154	0.999977237	Not Significant
Gm17660	2.638400562	2.159285319	1.086757292	1.986906677	0.04693274	0.999977237	up
Prkag2os1	16.76401578	1.030319882	0.518650857	1.986538474	0.046973565	0.999977237	up
Gm48627	7.032812986	1.134852099	0.571326452	1.98634615	0.046994902	0.999977237	up
Gm8213	2.638774881	2.131888575	1.073509037	1.985906499	0.047043707	0.999977237	up
Gm15749	1.351230701	-2.993650348	1.507533179	-1.985794	0.047056202	0.999977237	down
Rsad2	79.70428674	-0.65446226	0.329593041	-1.98566771	0.047070232	0.999977237	Not Significant
Elovl5	1848.959649	-0.242337434	0.122054183	-1.98549062	0.047089913	0.999977237	Not Significant
Gm15832	85.54374219	-0.349459167	0.176021965	-1.98531568	0.04710936	0.999977237	Not Significant
Gm19716	19.42604308	-0.848212362	0.427307486	-1.98501639	0.047142648	0.999977237	Not Significant

Gm45799	72.83791346	0.557824564	0.281263316	1.983282329	0.047335902	0.999977237	Not Significant
Mir670hg	123.1268067	0.321889164	0.162375851	1.982370911	0.047437743	0.999977237	Not Significant
Gm10062	1.40157413	3.107603158	1.568133068	1.981721591	0.047510409	0.999977237	up
ENSMUSG0000012147	121.4585963	-0.326781183	0.16506886	-1.97966584	0.047741089	0.999977237	Not Significant
6720468P15Rik	8.862385284	-1.101983606	0.556814008	-1.97908743	0.047806163	0.999977237	down
Pla2g6	525.6541337	-0.181326539	0.091633139	-1.97883147	0.047834984	0.999977237	Not Significant
Tmem117	121.7156093	-0.423277966	0.214155189	-1.9765011	0.048098051	0.999977237	Not Significant
ENSMUSG0000012129	9.673592975	-1.120926177	0.567216384	-1.97618794	0.048133495	0.999977237	down
Cthrc1	26.58419661	0.968321349	0.49039999	1.974554175	0.048318764	0.999977237	Not Significant
Gm31793	20.43725867	0.70117978	0.355308101	1.973441579	0.048445274	0.999977237	Not Significant
Prss53	64.82128969	-0.43889252	0.222691672	-1.97085287	0.048740707	0.999977237	Not Significant
Vmn1r60	15.04192121	0.926017944	0.470008682	1.970214551	0.048813787	0.999977237	Not Significant
Gm35368	25.62009506	-0.683504659	0.346950131	-1.97003718	0.048834109	0.999977237	Not Significant
Gm56127	3.205827933	2.476605299	1.257401473	1.969621758	0.048881736	0.999977237	up
Gm16235	4.800228485	-1.459205661	0.741309149	-1.96841718	0.049020057	0.999977237	down
Gm10643	47.37785823	0.550646691	0.279853315	1.967626116	0.049111073	0.999977237	Not Significant
Gm7340	32.85005693	0.601235442	0.30579411	1.96614461	0.049281909	0.999977237	Not Significant
Gm48054	55.40275466	0.450575054	0.229279562	1.965177573	0.049393689	0.999977237	Not Significant
Gm11434	1.364157527	3.081046185	1.56862867	1.964165416	0.049510912	0.999977237	up
Or14a260	13.96205905	-0.852803393	0.434216155	-1.9640066	0.049529327	0.999977237	Not Significant
Gbp10	8.904634591	-1.157564324	0.58977512	-1.96272153	0.049678541	0.999977237	down
