# Supplementary material for: Inhibition of Hippo Signaling Through Ablation of Lats1 and Lats2 Protects Against Cognitive Decline in 5xFAD Mice via Increasing Neuronal Resilience Against Ferroptosis
Source: Aging Cell. 2025 Sep 9;24(11):e70218. doi: 10.1111/acel.70218 (PMC12611316; doi:10.1111/acel.70218)
Supplement: Supplementary file 2 — Table S1: acel70218‐sup‐0002‐TableS1.zip. [file ACEL-24-e70218-s002.zip › Supplementary Table 1.docx]

Supplementary Table 1. Up- and Down- regulated genes in FAD-LatsKO mice
